# Multi-omic signatures of atherogenic dyslipidaemia: pre-clinical target identification and validation in humans

**DOI:** 10.1186/s12967-020-02663-8

**Published:** 2021-01-06

**Authors:** Mariola Olkowicz, Izabela Czyzynska-Cichon, Natalia Szupryczynska, Renata B. Kostogrys, Zdzislaw Kochan, Janusz Debski, Michal Dadlez, Stefan Chlopicki, Ryszard T. Smolenski

**Affiliations:** 1 Department of Biochemistry, Faculty of Medicine, Medical University of Gdansk, 1 Debinki St, 80-211 Gdansk, Poland; 2 Jagiellonian Centre for Experimental Therapeutics (JCET), Jagiellonian University, 14 Bobrzynskiego St., 30-348 Krakow, Poland; 3 Department of Nutritional Biochemistry, Faculty of Health Sciences, Medical University of Gdansk, 7 Debinki St., 80-211 Gdansk, Poland; 4 Department of Human Nutrition and Dietetics, Faculty of Food Technology, University of Agriculture in Krakow, 122 Balicka St., 30-149 Krakow, Poland; 5 Mass Spectrometry Laboratory, Institute of Biochemistry and Biophysics, Polish Academy of Sciences, 5a Pawinskiego St., 02-106 Warsaw, Poland; 6 Chair of Pharmacology, Jagiellonian University Medical College, 16 Grzegorzecka St., 31-531 Krakow, Poland

**Keywords:** Dyslipidaemia, Atherosclerosis, Metabolome, Proteome, Pathological mechanisms, Serological biomarkers

## Abstract

**Background:**

Dyslipidaemia is a major risk factor for atherosclerosis and cardiovascular diseases. The molecular mechanisms that translate dyslipidaemia into atherogenesis and reliable markers of its progression are yet to be fully elucidated. To address this issue, we conducted a comprehensive metabolomic and proteomic analysis in an experimental model of dyslipidaemia and in patients with familial hypercholesterolemia (FH).

**Methods:**

Liquid chromatography/mass spectrometry (LC/MS) and immunoassays were used to find out blood alterations at metabolite and protein levels in dyslipidaemic ApoE^−/−^/LDLR^−/−^ mice and in FH patients to evaluate their human relevance.

**Results:**

We identified 15 metabolites (inhibitors and substrates of nitric oxide synthase (NOS), low-molecular-weight antioxidants (glutamine, taurine), homocysteine, methionine, 1-methylnicotinamide, alanine and hydroxyproline) and 9 proteins (C-reactive protein, proprotein convertase subtilisin/kexin type 9, apolipoprotein C-III, soluble intercellular adhesion molecule-1, angiotensinogen, paraoxonase-1, fetuin-B, vitamin K-dependent protein S and biglycan) that differentiated FH patients from healthy controls. Most of these changes were consistently found in dyslipidaemic mice and were further amplified if mice were fed an atherogenic (Western or low-carbohydrate, high-protein) diet.

**Conclusions:**

The alterations highlighted the involvement of an immune-inflammatory response system, oxidative stress, hyper-coagulation and impairment in the vascular function/regenerative capacity in response to dyslipidaemia that may also be directly engaged in development of atherosclerosis. Our study further identified potential biomarkers for an increased risk of atherosclerosis that may aid in clinical diagnosis or in the personalized treatment.

## Background

Atherosclerosis, a major contributor to cardiovascular diseases (CVDs), is a pathogenic process with a complex/multi-factorial nature [1, 2]. Its diverse causal factors that include age, male gender, smoking, high blood pressure, diabetes mellitus and, in particular, dyslipidaemia have been well recognized, although the underlying mechanisms of atherogenesis still remain not fully understood [3, 4]. The majority of studies on atherogenesis are focused on vascular plaque formation and structure [5], and the results are difficult to translate into preventive markers in routine diagnostics and provide incomplete information concerning the mechanisms. Therefore, the identification of novel blood biochemical markers would be highly useful not only for a better understanding of the mechanisms involved but also to improve early diagnosis, monitoring of atherosclerosis progression and treatment. Several of such molecules for risk-stratification in atherosclerosis have been proposed, such as high-sensitivity cardiac troponin T (hs-cTnT), C-reactive protein (CRP), soluble CD40 ligand (sCD40L), adiponectin, interleukin-18 (IL-18), matrix metalloproteinase-9 (MMP-9), docosahexaenoic acid, glutamine and tyrosine [6–8], and some are under validation for use in clinical and research settings [9, 10]; however, none of these parameters are based on a comprehensive assessment of metabolomic and proteomic indices and, therefore, their implementation into clinical practice may not be adequate in all possible scenarios. There is a justified need to determine novel molecular indicators based on a broader spectrum of targets that alone, or in combination with existing ones, will provide better knowledge of the operating mechanisms leading to disease progression and its accurate biomarkers.

Experimental models that reproduce the pathogenic steps of atherosclerosis beginning with dyslipidaemia could pave the way for the discovery of the pathomechanisms involved and, eventually, biomarkers [11, 12]. Double ApoE^−/−^ and LDLR^−/−^ knockout mice constitute a reliable model of atherogenesis that begins with dyslipidaemia leading to atherosclerotic plaques. This model resembles human pathophysiology of the disease, featured by endothelial dysfunction, vascular inflammation and appearance of advanced atherosclerotic plaques with their distribution within the vasculature and histological features similar to humans [13–15]. ApoE^−/−^/LDLR^−/−^ mice display some advantages over single ApoE^−/−^ and LDLR^−/−^ knockouts as dyslipidaemia is evident even with a standard diet, and spontaneous atherosclerosis develops more rapidly [16]. Therefore, the research of risk factors related to atherosclerosis and its pathogenesis could benefit from the study of this model.

Recent technological advances, together with bioinformatics defined as the ‘omics’-based approaches (i.e., genomics, transcriptomics, proteomics and metabolomics), enable global characterization, at multiple levels (molecules/entire pathway), of complex biological systems and their changes in pathological processes [10, 17]. Among them, metabolomics and proteomics represent the most promising tools for the discovery of novel serological biomarkers and increasing understanding of the complex processes in atherosclerosis development/progression involved [18–23]. The number of reports using non-targeted or targeted metabolomics or proteomics for the study of CVDs is ever increasing, but most of them with only few exceptions [24, 25] concentrate on a single-omics technique, while a comprehensive dataset of proteins as well as metabolites participating in a given disease is urgently needed [26–28].

In this work, an integrated metabolomic and proteomic analysis was performed using multi-platform analytical tools to identify the molecular signature of atherogenic dyslipidaemia in ApoE^−/−^/LDLR^−/−^ mice. The metabolic/proteomic pathways related to biomarkers proposed were further explored to indicate possible targets for therapeutic intervention or prevention. That analysis was also followed by an investigation in patients with familial hypercholesterolemia (FH) and control subjects to validate the discrimination performance of the selected molecules and confirm their clinical relevance.

## Methods

### Animals and experimental design

ApoE/LDLR double knockouts on a C57BL/6 background (ApoE^−/−^/LDLR^−/−^) and wild-type C57BL/6 (WT) female mice, originally obtained from the Jackson Laboratory (JAX, USA), were bred in house and at the age of 6–7 months used in this study (Additional file 1: Table S1). The animals were housed in a 12-h day-night cycle in environmentally controlled rooms and allowed free access to water and a standard chow diet. Whole blood was collected via cardiac puncture into chilled tubes (containing EDTA as an anticoagulant) and centrifuged (4000×*g*, 10 min) to obtain plasma samples. The samples were analysed for: (a) proteomic pattern changes using a mass spectrometry (MS)-based non-targeted approach, (b) metabolomic pattern changes using an MS-based targeted approach and (c) systemic alterations within the renin-angiotensin system (RAS).

**Fig. 1:**
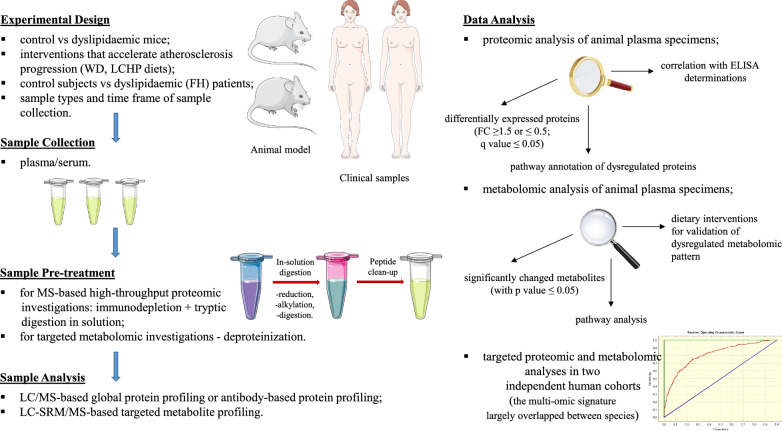
A flow diagram of multi-omic analysis was used to present the overview of the study design, how the experiments were performed, as well as the way of data treatment

For the survival experiment or evaluation of the effect of pro-atherogenic diets on plasma metabolomic pattern (to verify whether specific changes will be further accelerated along with atherosclerosis progression), the mice were fed a commercial, cholesterol-free, pelleted diet (Sniff M-Z Spezialdiäten GmbH; Soest, Germany) up to the age of 16–18 weeks. At this age, mice were weight-matched between groups (*n* = 42–49 (survival experiment) and *n* = 5–6 (metabolomics study) in each group) and fed AIN-93G-based diets: control diet (AIN) and experimental-Western or low-carbohydrate, high-protein (LCHP) diets for the next 8 weeks. Detailed compositions of the diets are provided in Additional file 1: Table S2, whereas the effects of dietary treatments on body and organ weight, as well as plasma biochemical markers, are presented in Additional file 1: Table S3. After 8 weeks of feeding (metabolomics study as well as an assessment of aortic atherosclerotic lesions), the mice were anaesthetized with ketamine/xylazine (100/10 mg/kg body weight) given intraperitoneally and sacrificed by cervical dislocation. Blood samples were taken under 4-h restricted feeding conditions from the left ventricle of the heart. A relevant flow diagram presenting the study design together with the experimental approach and multi-dimensional data analysis is shown in Fig. 1.

All procedures involving animals were conducted according to the ‘Guide for the Care and Use of the Laboratory Animals’ published by the European Parliament, Directive 2010/63/EU and were approved by the local Ethical Committees at the Medical University of Gdansk and the University of Agriculture in Krakow.

### Proteomic analysis of plasma

Plasma samples collected from knockout (*n* = 6) and control (*n* = 6) mice were pre-treated with an Aurum™ Serum Protein Mini Kit (Bio-Rad Laboratories, CA, USA) according to the manufacturer’s instructions. Proteins obtained by this enrichment method were subjected to a standard procedure of in-solution tryptic digestion followed by quantitative proteomics experiments using a well-validated label-free MS-based method [29–31].

The resulting peptide mixtures were applied onto an RP-C_18_ trapping column (20 mm × 180 µm, 5 µm; Waters, Milford, USA) of the nanoUPLC system using 0.1% (v/v) formic acid in water as the mobile phase and then transferred to a nanoACQUITY UPLC BEH C_18_ analytical column (250 mm × 75 µm, 1.7 µm; Waters, USA) using a 160-min linear gradient of 0–35% acetonitrile (ACN) at a flow rate of 250 nL/min. The column outlet was directly coupled to the nanoelectrospray (nanoESI) ion source of the LTQ-Orbitrap Velos mass spectrometer (Thermo Scientific, San Jose, USA). Each sample was measured in duplicate—once for protein sequencing (data-dependent MS to MS/MS switch) and once for quantitative information (MS only, sequencing disabled). Full MS spectra were collected at a resolution of 60,000 (for ions at m/z 400) over the range of m/z 300–2000. The ten most abundant precursor ions were selected for fragmentation, which was achieved using higher-energy collisional dissociation (HCD) with a normalized collision energy setting of 35%. The MS/MS spectra were collected at a resolution of 15,000 (m/z 400) and parent ion activation was performed with an isolation width of 2 Da and a minimal intensity of 5,000 counts. Further details regarding the analytical conditions that were applied can be found in [29, 30].

The acquired MS/MS data were pre-processed with Mascot Distiller software (v. 2.3, Matrix Science, London, UK), and the search was carried out with the Mascot Search Engine (Matrix Science, Mascot Server 2.4) in a three-step procedure described elsewhere [29] against the set of *Mus Musculus* protein sequences derived from the Uniprot database (total 74,459 proteins), merged with a randomized version. The initial search parameters were set as follows: 20 ppm tolerance for precursor ion masses; 0.4 Da for fragment ion masses; a maximum of two missed tryptic cleavages; fixed modification: carbamidomethylation (C); variable modifications: oxidation (M), acetyl (peptide N-term) and deamidation (N, Q). A statistical assessment of peptide assignments was built on the concatenated target/decoy database search strategy and a procedure that provided the *q*-values for each peptide spectrum match (PSM). Only PSMs with *q*-values of ≤ 0.01 and proteins represented by at least two peptides were regarded as confidently identified. The list of peptides meeting the aforementioned acceptance criteria and unique for proteins' identification were subjected to a step of quantitative analysis to obtain a list of proteins differentially expressed between a set of ApoE^−/−^/LDLR^−/−^ and WT mice. A more detailed description of the MS-based proteomic procedures is available in our previous work [31].

### Confirmation of plasma protein variations by ELISA analysis

Immunoassays-based quantitations of serum amyloid A-1 protein (SAA1), proprotein convertase subtilisin/kexin type 9 (PCSK9), soluble vascular cell adhesion molecule-1 (sVCAM-1), fibrinogen (FBG), angiotensinogen (AGT) and glutathione peroxidase 3 (GPx-3) were all performed using commercial kits according to the manufacturer’s protocols (Abcam, Cambridge, UK). Of note, alterations in AGT level were evaluated because results from untargeted runs indicated that the expression of enzymes responsible for its degradation/conversion was largely up-regulated, suggesting massive over-activation of RAS.

### Metabolomic analysis of plasma

Metabolomic profiling of plasma was performed according to the protocol described previously [31]. Briefly, aliquots of 50 µL of plasma samples were pipetted into 2-mL vials, followed by the addition of 10 μL of the internal standard solution and ACN at the proportion of 2:1 (v/v) into each vial. The tubes were then centrifuged at 4 °C, 16,000×*g* for 15 min, and the resultant supernatant was collected and subjected to freeze-drying. The residue was next reconstituted with 50 μL of 0.1% formic acid in water and analysed using an LC–MS/MS system as detailed below.

Simultaneous determination of 44 underivatized amino acids (AAs) in mouse plasma was performed using a Surveyor HPLC system (Thermo Scientific, San Jose, CA, USA) coupled to a TSQ Vantage Triple-Stage Quadrupole mass spectrometer (Thermo Scientific) that was equipped with a heated electrospray ionization probe (HESI-II). The chromatographic separation was achieved on a Synergi Hydro-RP column (50 mm × 2.0 mm, 2.5 µm; Phenomenex, Torrance, CA, USA) maintained at 25 °C. The mobile phase consisted of deionized water (A) and ACN (B), containing 5 mM nonafluoro-1-pentanoic acid (to improve the retention of polar analytes, A) and 0.1% formic acid (B), respectively, and the elution gradient used was as follows: 0 min, 0% B; 2 min, 30% B; 4.5 min, 45% B; 4.5–9 min 0% B, resulting in a total run time of 9 min. The flow rate was 0.2 mL/min, and the injection volume was 2 µL. Data acquisition was carried out in selected reaction monitoring (SRM) mode employing electrospray ionization (ESI) in positive mode. Analytical method details, as well as the MS/MS transitions monitored in the protocol, are described elsewhere [31]. Instrument control, data collection and analysis were achieved using Thermo Xcalibur 2.1 software.

### Systemic and tissue angiotensin (Ang) profile determination

Circulating and aortic Ang peptide concentrations were determined by mass spectrometry linked to liquid chromatography using plasma/tissue samples collected in the presence of a protease inhibitor cocktail that completely blocked Ang metabolism, as described previously [32]. Stabilized protease inhibitor samples (25 µL) were further spiked with 5 µL internal standard solution ([Asn^1^, Val^5^]-Ang II) at a concentration of 250 pg/mL and deproteinized with ACN (in 4:1 (v/v) proportion to the samples used). Following C18-based solid-phase-extraction, samples were subjected to LC–MS/MS analysis using a reversed-phase analytical column (Acclaim PepMap100 RSLC C18, 150 mm × 75 µm, 2 µm) of the UltiMate 3000 Rapid Separation nanoLC system operating in line with a TSQ Vantage triple quadrupole mass spectrometer (Thermo Scientific). Separation of the peptides was performed by gradient elution using a mobile phase comprising water (solvent A) and ACN (solvent B), both containing 1% acetic acid (v/v). The flow rate was 300 nL/min, and the overall chromatographic run time including re-equilibration was 50 min. For the MS detection, the instrument was operated in the positive ESI mode, and the acquisition was performed in SRM mode. The two most sensitive/specific ion transitions were measured for each of the nine Ang peptides determined [Ang-(1–12), Ang I, II, III, IV, (1–7), (1–9), Ang A and alamandine]. Optimum analytical conditions, as well as the precursor and product ions used in the protocol, were described previously [32]. The instrumental operation, data acquisition and peak integration were performed using Thermo Xcalibur 2.1 software.

### Familial hypercholesterolemia patients and controls

Serum samples of patients with FH diagnosed both clinically and genetically (under the care of the National Centre for Familial Hypercholesterolemia in Gdansk) and non-FH normolipidemic subjects as controls were used in this study. FH cases and controls were divided into discovery (*n* = 20 + 20) and validation (*n* = 29 + 29) cohorts. The participation of the study subjects was approved by the Bioethics Committee at the Medical University of Gdansk upon written informed consent. All procedures involving human participants were in accordance with the ethical standards of the 1964 Helsinki declaration and its later amendments. FH patients and healthy control subjects were matched for age, sex and body mass index (BMI). Subjects’ clinical characteristics are compiled in Additional file 1: Tables S4 and S5.

### Analysis of protein and metabolite signatures in human sera

Serum concentrations of candidate proteins: C-reactive protein (CRP), PCSK9, apolipoprotein C-III (ApoC-III), soluble intercellular adhesion molecule-1 (sICAM-1), AGT, paraoxonase/arylesterase 1 (PON-1), fetuin-B (FETUB), vitamin K-dependent protein S (VKDP-S) and biglycan (BGN) (Abcam, Cambridge, UK; Uscn, Life Science Inc. USA) were determined in individual controls and patient subjects using ELISA kits according to the manufacturer’s protocols.

For metabolite analysis, serum protein removal was performed by precipitation in pre-chilled ACN, and the resultant supernatant was collected and subjected to freeze-drying. After reconstitution in 0.1% formic acid in Milli-Q water, samples were analysed by (SRM)-LC‐MS/MS as described in the previous paragraph.

### Statistical and pathway analyses

Data are presented as mean ± SEM unless otherwise noted. Statistical differences were determined using Student’s unpaired *t* test (when two groups were compared) or one-way ANOVA with a Bonferroni post hoc test (when more than two groups were compared). Comparisons resulting in *p*-values lower than 0.05 were considered statistically significant. Statistical analyses were performed using Prism 9.0 (GraphPad Software, Inc., La Jolla, CA, USA).

Protein–protein interaction analysis of the differentially expressed proteins was performed using STRING 11.0 software with the following analysis parameters: species—*Mus musculus*, confidence level − 0.4 and active prediction methods-all. This freely available database of known and predicted protein interactions provides information on associations derived from genomics, high-throughput experiments, co-expression and previous knowledge. Metabolic pathway analysis (MetPA) was performed using MetaboAnalyst 4.0 (http://www.metaboanalyst.ca/) to identify potentially affected metabolic pathways. This web tool conducts pathway analysis through pathway enrichment and topological analysis. In this study, we selected the *Mus musculus* library and used the default ‘Hypergeometric Test’ (for pathway enrichment analysis) and the ‘Relative-Betweenness Centrality’ algorithm (for pathway topological analysis). To identify the most relevant pathways, the impact-value threshold derived from pathway topology analysis was set to 0.1. A multivariate ROC (Receiver Operating Characteristic) curve based exploratory analysis was performed for the purpose of the most discriminative features’ selection, model building, and performance evaluation and that was achieved with MetaboAnalyst 4.0 web server (Biomarker Analysis module). The Random Forest for the classification and “Random Forest built-in” for the feature ranking method was employed as a multivariate algorithm to facilitate in selecting variables with the highest predictive accuracy. The survival rates among groups were estimated by the Kaplan–Meier method and compared using a log-rank test.

## Results

### Protein alterations in response to atherogenic dyslipidaemia

We first investigated the most significant protein changes in plasma of dyslipidaemic mice. Hence, differential protein expression analysis with LC/MS was performed using 6 specimens per group matched by age, gender and basal characteristics. Of a total of 523 unique proteins identified with a sufficient MS signal in the current study, 279 were detected in at least 4 samples per group, which constituted our threshold for inclusion in the statistical analysis (dataset provided in attached Additional file 2). Statistical analysis of the data revealed changes in the abundance of 47 proteins (*q* < 0.05): 34 up-regulated and 13 down-regulated in atherogenic dyslipidaemia (Table 1). The majority of these differentially expressed proteins could be classified into relevant functional categories, namely: (1) inflammation and immune response (serum amyloid A-1/A-2/A-3/A-4 protein, serum amyloid P-component, α-2-macroglobulin, α-1-antitrypsin 1-1/1-2/1-3, adiponectin, gelsolin, complement component C9, complement factor B, complement factor H, inter alpha-trypsin inhibitor, heavy chain 4), (2) lipid transport/metabolism (apolipoprotein A-IV, apolipoprotein B-100, apolipoprotein C-I, apolipoprotein C-III, proprotein convertase subtilisin/kexin type 9), (3) adhesion of leukocytes to endothelial cells (ECs)/endothelial activation (vascular cell adhesion protein 1, laminin subunit gamma-1, thrombospondin-4, angiopoietin-1), (4) blood coagulation/fibrinolysis (von Willebrand factor, coagulation factor VII, vitamin K-dependent protein S, fibrinogen beta chain, kininogen-1, plasma kallikrein), (5) RAS (renin-angiotensin system) over-activation (angiotensin-converting enzyme (ACE), cathepsin D), (6) detoxification of reactive oxygen species (ROS) (catalase, serum paraoxonase-1, glutathione peroxidase 3), (7) extracellular matrix reorganization/arterial remodelling (tripeptidyl-peptidase 1, EGF-containing fibulin-like extracellular matrix protein 1, lumican, biglycan, fetuin-B, transforming growth factor beta-1), and (8) haeme clearance (hemopexin, serotransferrin).

**Table 1:** Most significant proteins differentially expressed in plasma of ApoE^−/−^/LDLR^−/−^ vs. wild-type mice as revealed by proteomic analysis (the ratio is given as ApoE^−/−^/LDLR^−/−^**/**control)

No	UniProtKB ID/Accession number	Q value	Ratio	Peptide count	Protein annotation (Short name)	Involvement in the biological process
1	P15535	N/A*	Only in 2^#^	2	Beta-1,4-galactosyltransferase 1 (Beta-1,4-GalTase 1)	*Protein glycosylation*
2	P07310	N/A	Only in 1^#^	2	Creatine kinase M-type (M-CK)	*Energy transduction/Creatine metabolism*
3	P29533	N/A	Only in 1	2	Vascular cell adhesion protein 1 (VCAM-1)	*Leukocyte*-*endothelial cell adhesion*
4	P02468	N/A	Only in 1	2	Laminin subunit gamma-1 (S-LAM gamma)	*Cell adhesion*
5	P24270	N/A	Only in 2	2	Catalase (Cat)	*Detoxification of Reactive Oxygen Species*
6	O89023	N/A	Only in 1	2	Tripeptidyl-peptidase 1 (TPP-1)	*Peptide catabolic process/Vessels wall injury*
7	P63017	N/A	Only in 1	2	Heat shock cognate 71 kDa protein (Hspa8)	*Molecular chaperone implicated in a wide variety of cellular processes associated with proteome organization*
8	P04918	N/A	Only in 1	5	Serum amyloid A-3 protein (SAA3)	*Acute*-*phase response*
9	P06728	0.00003	0.57	64	Apolipoprotein A-IV (ApoA-IV)	*Lipid transport/metabolism*
10	P13020	0.00003	0.00003	31	Gelsolin (GSN)	*A key component of the innate immune system*
11	E9Q414	0.00003	1.44	339	Apolipoprotein B-100 (ApoB-100)	*Lipid transport/metabolism*
12	P05366	0.00003	3.13	11	Serum amyloid A-1 protein (SAA1)	*Acute*-*phase response*
13	P05367	0.00003	3.34	15	Serum amyloid A-2 protein (SAA2)	*Acute*-*phase response*
14	A6X935	0.00003	1.38	72	Inter alpha-trypsin inhibitor, heavy chain 4 (ITI-HC4)	*Acute*-*phase response/Hyaluronan metabolic process*
15	Q61838	0.00073	0.82	81	Alpha-2-macroglobulin (Alpha-2-M)	*Disruption of inflammatory cascade/Serine protease inhibition*
16	Q921I1	0.00077	0.43	23	Serotransferrin (Transferrin/STF)	*Iron transport*
17	P07309	0.00082	0.71	9	Transthyretin (TTR)	*Retinoid metabolism and transport*
18	P12246	0.00367	2.55	10	Serum amyloid P-component (SAP)	*Acute*-*phase response*
19	P06683	0.00377	1.44	17	Complement component C9 (C9)	*Complement alternate pathway*
20	Q91X72	0.00489	0.58	22	Hemopexin (HPX)	*Scavenging of heme from plasma*
21	P31532	0.01109	21.671	14	Serum amyloid A-4 protein (SAA4)	*Acute*-*phase response*
22	P07758	0.01969	1.46	14	Alpha-1-antitrypsin 1-1 (Serpina 1a)	*Acute*-*phase response*
23	Q00896	0.01969	1.39	14	Alpha-1-antitrypsin 1-3 (Serpina 1c)	*Acute*-*phase response*
24	P04186	0.02333	1.35	17	Complement factor B (CFB)	*Complement alternate pathway*
25	P22599	0.03604	1.44	6	Alpha-1-antitrypsin 1-2 (Serpina 1b)	*Acute*-*phase response*
26	Q80W65	0.03649	1.83	11	Proprotein convertase subtilisin/kexin type 9 (PCSK9)	*Cholesterol metabolism*
27	Q8BPB5	0.03766	0.73	7	EGF-containing fibulin-like extracellular matrix protein 1 (FIBL-3)	*A growth factor in the arteries*
28	P51885	0.03795	1.58	17	Lumican (LUM)	*Collagen fibril organization*
29	P06909	0.03892	1.31	23	Complement factor H (CFH)	*Complement alternate pathway*
30	P28653	0.03905	1.95	13	Biglycan (BGN)	*Blood vessel remodelling*
31	Q60994Q60994	0.039430.03943	0.45	6	Adiponectin (AdipoQ/APN)	*Negative regulation of tumor necrosis factor*-*mediated signaling pathway*
32	P70375	0.04003	1.55	9	Coagulation factor VII (FVII)	*Blood coagulation*
33	Q08761	0.04005	0.79	12	Vitamin K-dependent protein S (VKDP-S)	*Prevention of coagulation/Fibrinolysis stimulation*
34	Q9Z1T2	0.04106	1.47	2	Thrombospondin-4 (TSP-4)	*Endothelial cell*–*cell adhesion/Tissue remodelling*
35	Q8K0E8	0.04213	1.62	10	Fibrinogen beta chain (FGB)	*Blood coagulation*
36	Q9QXC1	0.04378	1.54	4	Fetuin-B (FETUB)	*Regulation of vascular plaque*-*stabilizing factors*
37	O08677	0.04451	1.29	19	Kininogen-1 (KNG1)	*Blood coagulation*
38	P09470	0.04568	1.66	28	Angiotensin-converting enzyme (ACE)	*Angiotensin II generation/Bradykinin inactivation*
39	P26262	0.04571	1.48	11	Plasma kallikrein (pKal)	*Blood coagulation/Inflammatory response*
40	O08538	0.04864	1.62	8	Angiopoietin-1 (ANG-1)	*Regulation of blood vessel endothelial cell migration/cell adhesion/monocyte recruitment and retention*
41	Q8CIZ8	0.04903	1.83	19	Von Willebrand factor (vWF)	*Blood coagulation/Cell adhesion/Platelet activation*
42	P34928	0.049540.04954	1.54	7	Apolipoprotein C-I (ApoC-I)	*Lipid transport/metabolism*
43	P18242	0.04966	1.38	4	Cathepsin D (Cath-D)	*Cleavage of angiotensinogen to produce angiotensin I/Renin*-*like activity*
44	P33622	0.04986	1.47	8	Apolipoprotein C-III (ApoC-III)	*Lipid transport/metabolism*
45	P04202	0.04997	1.44	8	Transforming growth factor beta-1 (TGF-beta-1)	*Controlling of proliferation, differentiation and cell migration/Induction of epithelial*-*to*-*mesenchymal transition*
^&^46	P52430	0.05003	0.58	25	Serum paraoxonase/arylesterase 1 (PON-1)	*Enzymatic protection of low*-*density lipoproteins against oxidative modification*
^&^47	P46412	0.05015	0.72	10	Glutathione peroxidase 3 (GPx-3)	*Detoxification of Reactive Oxygen Species*

* N/A – not applicable; # only in 1 refers to ApoE/LDLR^−/−^, whereas in 2 refers to controls

& These proteins were involved in the table (despite q-value slightly higher than 0.05) as they support biological conclusion about profoundly affected anti-oxidative defense mechanisms in response to dyslipidaemia

**Fig. 2:**
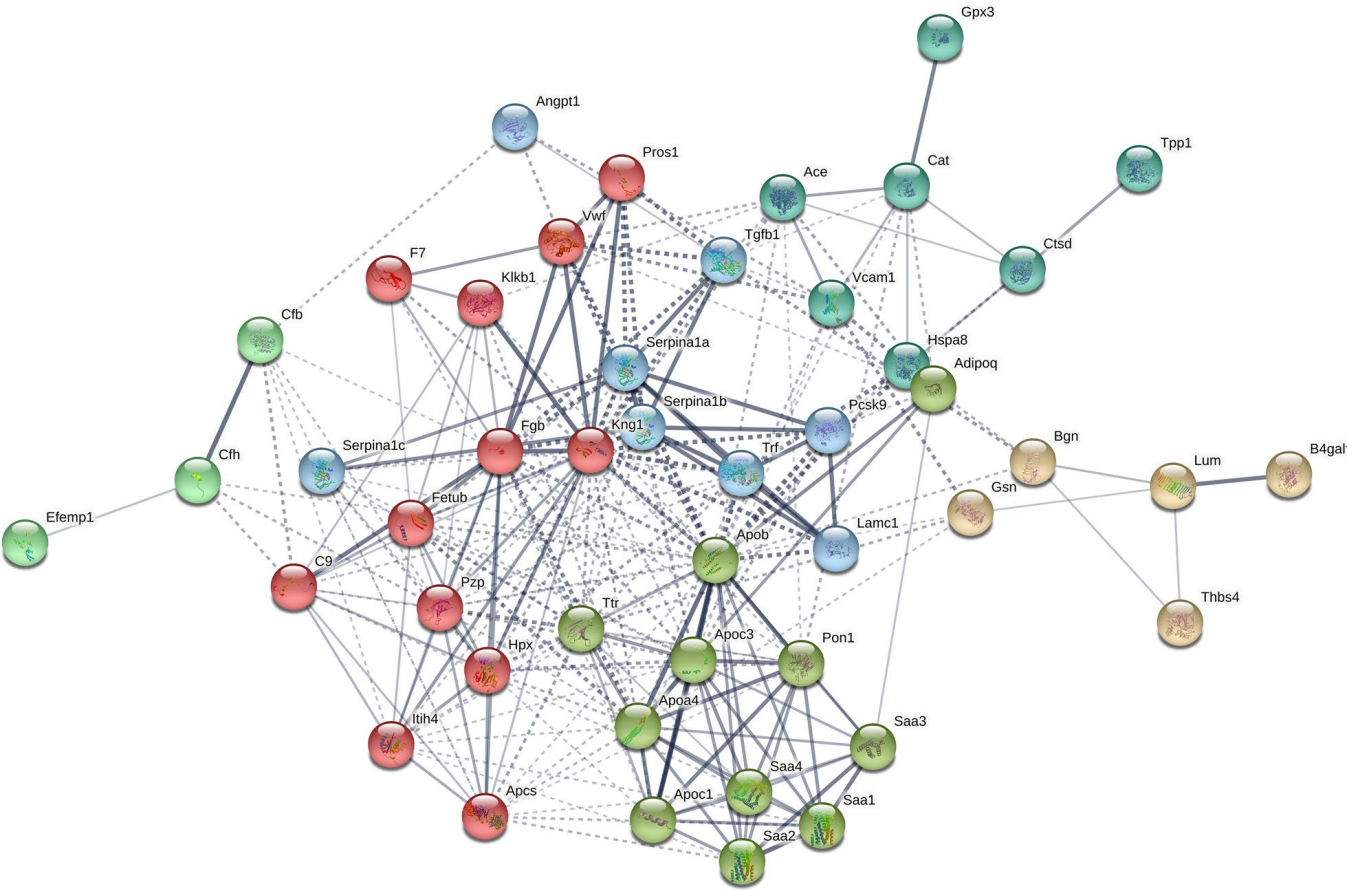
The protein–protein interactions for the differentially expressed proteins in ApoE^−/−^/LDLR^−/−^ mice (vs. WT) that were analysed using STRING 11 software. In the network analysis the differentially expressed proteins were presented as nodes, whereas edges represent predicted protein–protein associations. Using the protein interaction network analysis tool (STRING database), seven networks of the associated proteins were found among the differentially expressed proteins that were depicted by relevant colours. These included proteins mainly involved in: (1) acute phase response and/or being a constituent of lipoprotein particles (olive), (2) coagulation cascade (red), (3) alternative complement pathway (mint), (4) existing as inhibitors of serine proteases (light blue), (5) participating in adhesion of leukocytes to endothelial cells/renin-angiotensin system over-activation or modulation of oxidative stress (turquoise), (6) modulation of extracellular interactions/extracellular matrix re-organization (salmon), and (7) others – the disconnected node in the network represented by creatine kinase (hidden in the plot)

To better understand the nature of the differentially expressed proteins and how these proteins interact with each other, we searched the String database for known and predicted protein associations against the proteins we identified. Figure 2 presents constructed protein–protein interaction networks. Further, to validate the results obtained by the label-free MS-based method, six representative proteins (SAA1, PCSK9, sVCAM-1, FBG, AGT and GPx-3) reflecting the diverse nature of biochemical processes in which they participated were selected for immunochemical analyses. In these analyses, SAA1, PCSK9, sVCAM-1, FBG and AGT were found to be up-regulated in plasma of ApoE^−/−^/LDLR^−/−^ mice (vs WT), whereas GPx-3 was down-regulated, which is consistent with the results obtained from the non-targeted proteomics approach (Additional file 1: Figure S1).

### Metabolic alterations in response to atherogenic dyslipidaemia

**Fig. 3:**
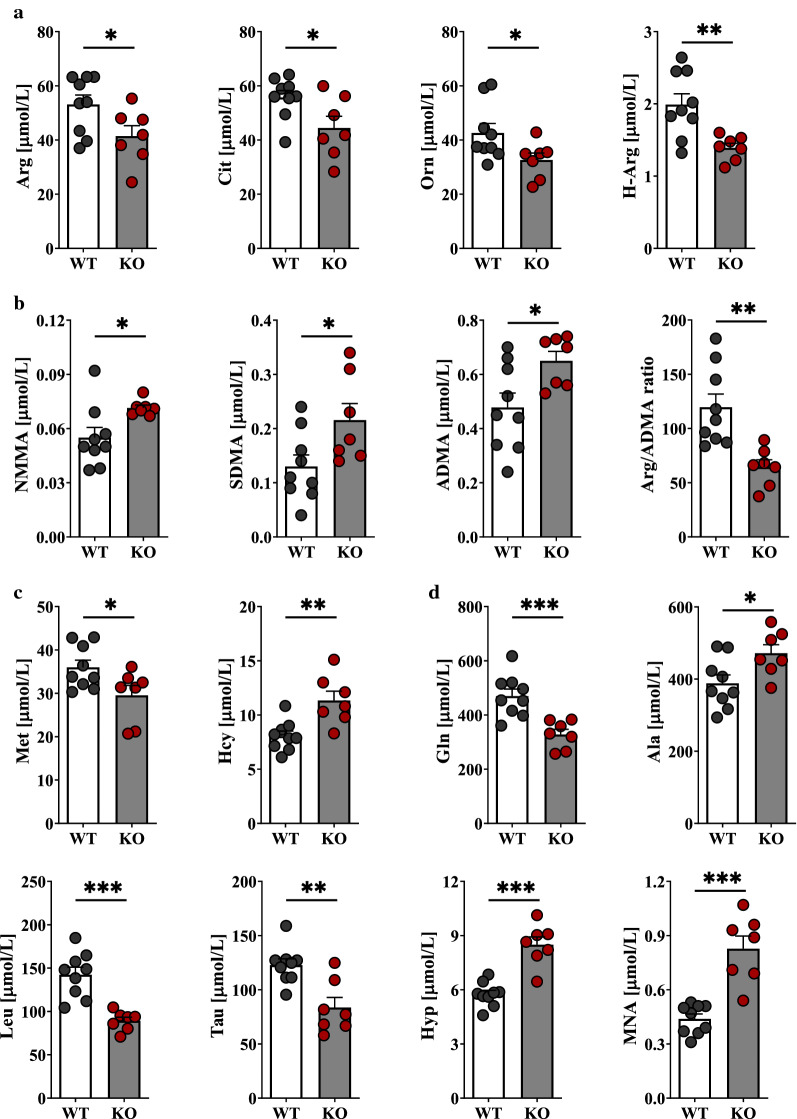
Significantly altered metabolites in response to atherogenic dyslipidaemia in ApoE^−/−^/LDLR^−/−^ mice. Plasma concentration of substrates for NOSs (L-Arg, H-Arg) and Arg precursors (L-Cit, L-Orn) (**a**), methylated Arg derivatives (NMMA, SDMA, ADMA) as well as L-Arg/ADMA ratio (**b**), L-Met, Hcy (**c**) and other metabolites significantly changed in response to dyslipidaemia (**d**) in ApoE^−/−^/LDLR^−/−^ (*n* = 7) and WT (*n* = 9) mice. Data represent mean ± SEM. *P < 0.05, **P < 0.01, ***P < 0.001 vs. WT

**Fig. 4:**
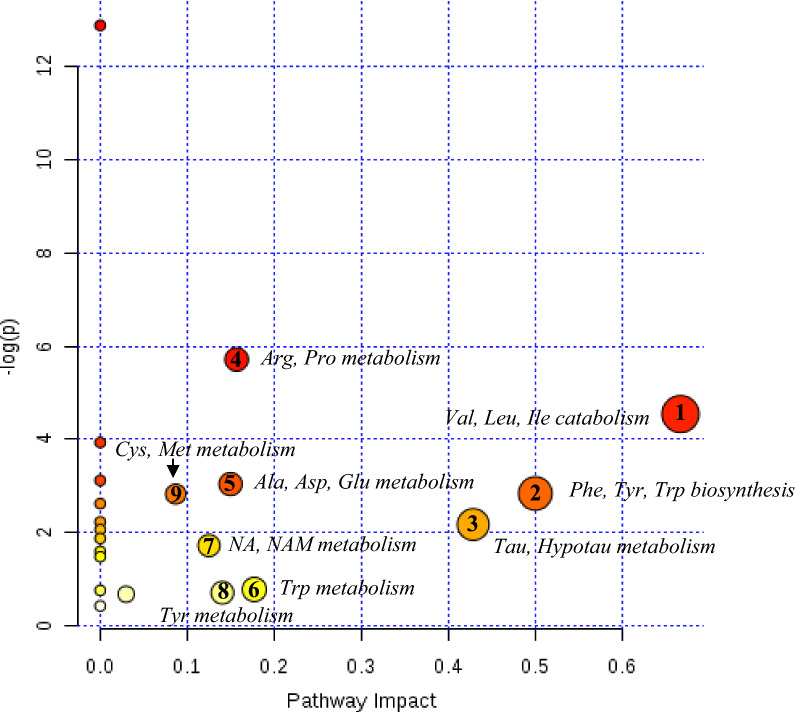
Summary of altered metabolic pathways analysed by MetaboAnalyst 4.0 web software. As shown, nine metabolic pathways of importance were disturbed in the plasma of dyslipidaemic mice (as compared to the wild-types): (1) valine, leucine and isoleucine degradation; (2) phenylalanine, tyrosine and tryptophan biosynthesis; (3) taurine and hypotaurine metabolism; (4) arginine and proline metabolism; (5) alanine, aspartate and glutamate metabolism; (6) tryptophan metabolism; (7) nicotinate and nicotinamide metabolism; (8) tyrosine metabolism, and (9) cysteine and methionine metabolism. Significantly affected pathways are plotted according to *P*-value from pathway enrichment analysis and pathway impact score from pathway topology analysis. Circle size and colour gradient indicate the significance of the pathway ranked by *P*-value (red: lower *P*-values and yellow: higher *P*-values) and pathway impact score (the larger the circle the higher the impact score), respectively

To investigate the metabolic changes that occurred in plasma in response to atherogenic dyslipidaemia, plasma samples (ApoE^−/−^/LDLR^−/−^ and WT) were explored by targeted LC–MS/MS. A total of 44 compounds were quantified (Additional file 1: Table S6), among which amino group-containing metabolites represented the most important subset of the metabolome. These metabolites included proteinogenic and non-proteinogenic amino acids carrying amino and acidic (e.g., carboxyl or sulfonic) groups, post-translationally modified (e.g., methylated) amino acids, small peptides, thiol and disulphide containing amino metabolites. Because they cover dozens of important metabolic pathways, quantitative analysis of these metabolites is, therefore, critical for biomarker discoveries. Twenty-four metabolites were significantly altered (*p*-value < 0.05) in ApoE^−/−^/LDLR^−/−^ mice compared with the wild types. Among these metabolites, the plasma levels of 6 metabolites, asymmetric (ADMA) and symmetric (SDMA) dimethylarginines, homocysteine (Hcy), 1-methylnicotinamide (MNA), 5-hydroxylysine (Hyl) and 4-hydroxyproline (Hyp) increased strikingly in dyslipidaemic mice compared with the controls, whereas the levels of 3 other (alanine (Ala), creatinine (Cre) and monomethylarginine (NMMA)) metabolites accumulated markedly. In contrast, urea cycle-related AAs (i.e., arginine (Arg), citrulline (Cit), ornithine (Orn) and glutamine (Gln)) and branched-chain AA (BCAA)-related AAs (isoleucine (Ile), leucine (Leu)), as well as taurine (Tau) and methionine (Met), were down-regulated in pathological samples. In addition, the plasma L-Arg/ADMA ratio and homoarginine (H-Arg) concentration—the indicators for NO bioavailability—were decreased with atherogenic dyslipidaemia. A total of 15 metabolites with the most altered plasma features were selected and are presented in Fig. 3. Metabolic pathway analysis revealed nine pathways involved in response to dyslipidaemia (Fig. 4).

### Systemic and local RAS alterations in ApoE^−/−^/LDLR^−/−^ mice

We compared the plasma and aortic levels of nine Ang peptides in the pathological and normal state (Additional file 1: Figure S2). Circulating levels of Ang I and Ang II, as well as other pathological effectors of the RAS (Ang III, IV, A), were significantly elevated in dyslipidaemic mice compared with the wild types, whereas the increase in Ang-(1–12) was modest. These changes were associated with a decrease in plasma Ang-(1–7) concentration and Ang-(1–7)/Ang II ratio (in ApoE^−/−^/LDLR^−/−^ mice). In the aorta, the pattern of changes was largely convergent with that observed in plasma, pointing to an over-activation of the ACE/Ang II pathway and down-regulation of the ACE-2/Ang-(1–7) pathway. Interestingly, the low levels of Ang-(1–10) at relatively high Ang-(1–12) could suggest the existence of an alternative pathway of Ang II generation that may be dependent on chymase in the aorta.

### Effect of pro-atherogenic diets on plasma AA metabolome

**Fig. 5:**
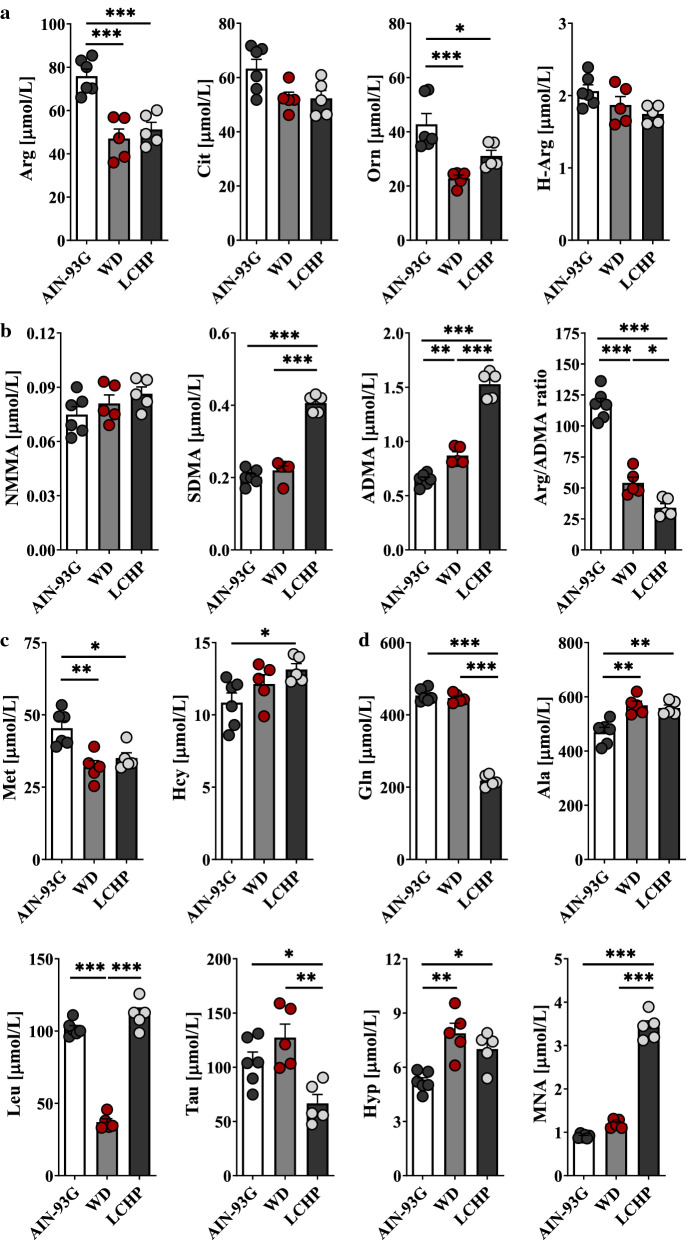
Effect of pro-atherogenic diets (Western and low-carbohydrate, high-protein (LCHP) diets) on plasma AA (amino acid-related) metabolome in ApoE^−/−^/LDLR^−/−^ mice. Plasma concentration of substrates for NOSs (L-Arg, H-Arg) and Arg precursors (L-Cit, L-Orn) (**a**), methylated Arg derivatives (NMMA, SDMA, ADMA) as well as L-Arg/ADMA ratio (**b**), L-Met, Hcy (**c**) and other metabolites related to atherosclerosis progression (**d**) in 6-month-old ApoE^−/−^/LDLR^−/−^ mice fed for 2 months: Control-AIN-93G (*n* = 6), WD (*n* = 5) or LCHP (*n* = 5) diet, respectively. Data represent mean ± SEM. *, **, and *** indicate *P* < 0.05, *P* < 0.01, *P* < 0.001, respectively

Dietary treatments (LCHP (low-carbohydrate, high-protein) or Western diet) promoted atherosclerosis in ApoE^−/−^/LDLR^−/−^ mice (Additional file 1: Fig. S3) [33] and had a significant impact on plasma metabolomic pattern (Additional file 1: Table S7). LCHP diet accelerated the disease more intensively than the classical Western diet and favoured the development of unstable lesions (Additional file 1: Fig. S3) [33, 34] that translated into more profound metabolic alterations as compared with WD. The plasma levels of metabolites that initially were significantly elevated in pathological samples (i.e., ADMA, SDMA, Hcy, MNA, Hyp, Ala) further increased with WD or LCHP feeding. A massive increase in the concentration of dimethylarginines and MNA was a dominant feature of the plasma metabolite profile of animals fed the LCHP diet as compared to other (AIN-93G or Western) diets. Surprisingly, the increase in MNA was as much as fourfold in LCHP-fed ApoE^−/−^/LDLR^−/−^ in comparison to mice fed the control diet. By contrast, the levels of urea cycle-related AAs, as well as Met and Tau, were diminished with LCHP feeding. Interestingly, plasma creatinine concentration significantly decreased in ApoE^−/−^/LDLR^−/−^ on atherogenic diets; this result may indicate a compensatory increase in creatinine clearance in these animals. Figure 5 presents the top 15 most important metabolite features identified.

### Translational study in patients with familial hypercholesterolemia

**Fig. 6:**
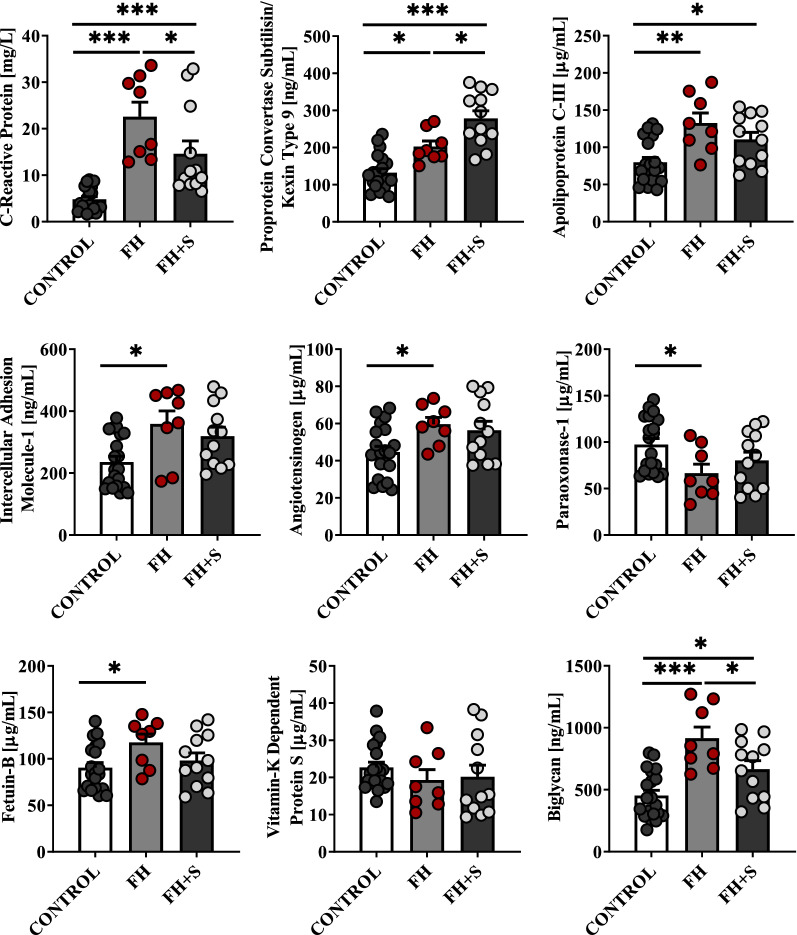
Translational study in human sera coming from patients affected by FH (*n* = 20) and healthy subjects (*n* = 20) (discovery cohort). Quantification of selected marker candidates (CRP, PCSK9, ApoC-III, sICAM-1, AGT, PON-1, FETUB, VKDP-S, and BGN) in crude serum samples by colorimetric ELISA. Protein levels are presented as mean ± SEM. *, **, and *** indicate *P* < 0.05, *P* < 0.01, *P* < 0.001, respectively. *FH *+ *S* – FH patients receiving statin therapy

**Fig. 7:**
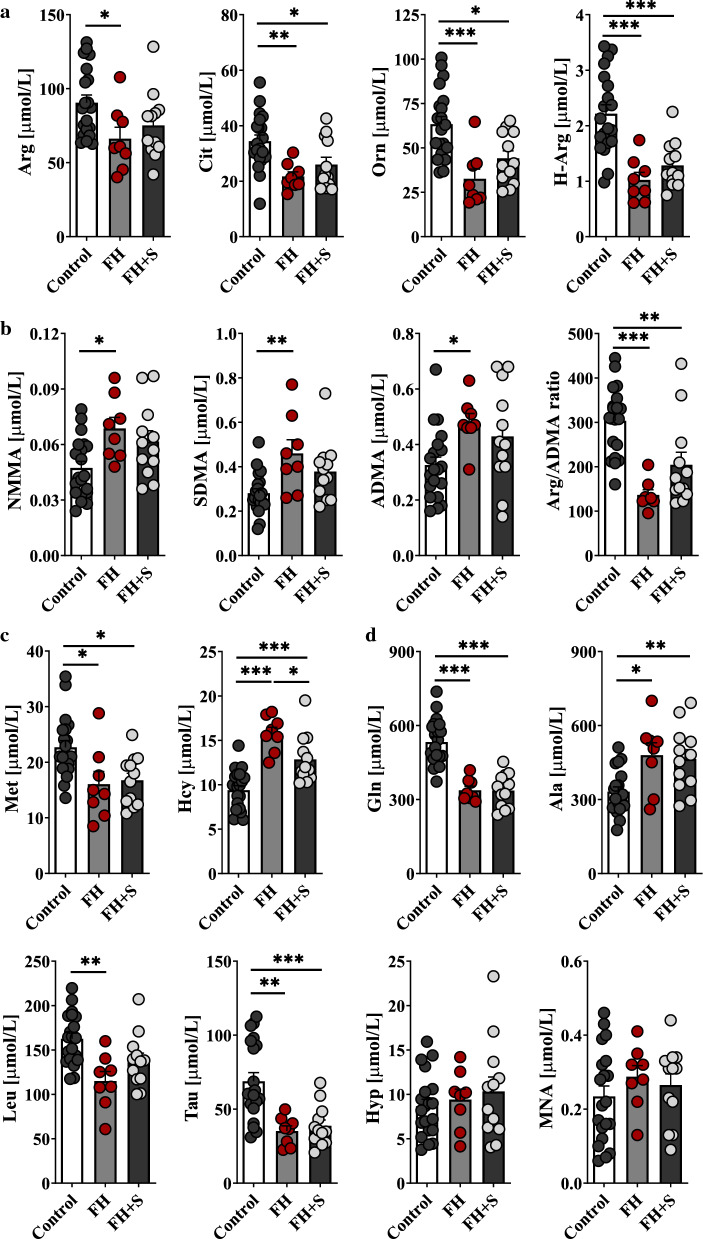
A snapshot of serum metabolites in the setting of familial hypercholesterolemia (discovery cohort). Human metabolomic signatures related to atherogenic dyslipidaemia closely reflect the pattern already identified in ApoE^−/−^/LDLR^−/−^ mice. The top (**a**) row depicts serum concentration of substrates for NOSs (L-Arg, H-Arg) and Arg precursors (L-Cit, L-Orn), the middle rows -methylated Arg derivatives (NMMA, SDMA, ADMA) and L-Arg/ADMA ratio (**b**), and L-Met, Hcy (**c**), respectively and **d** refers to other altered metabolites determined in FH cases (*n* = 20) vs. healthy subjects (*n* = 20). Data represent mean ± SEM. *P < 0.05, **P < 0.01, ***P < 0.001. *FH *+ *S* – FH patients on statins

To translate our pre-clinical findings into a human setting, we also characterized molecular alterations in human serum samples. A distinct set of proteins for comparison was selected based on initial conclusions received for animal samples that were subjected to comparative global blood plasma protein profiling. Only some minor changes (in the pattern of proteins’ determined) were implemented based on the literature or our previous observations: (1) sVCAM-1 was replaced by sICAM-1 because the latter proved to be more up-regulated in humans in response to hyperlipidaemia [35], (2) CRP level (instead of SAA) was analysed since it is more commonly used in clinical practice demonstrating comparable sensitivity as a measure of inflammation [36] and, finally, (3) alterations in AGT level were evaluated to verify whether dyslipidaemia also initiated profound RAS activation in human samples. Nine proteins (PCSK9, ApoC-III, sICAM-1, AGT, PON-1, FETUB, VKDP-S, BGN and CRP) were found significantly altered in sera from FH patients as compared to the healthy subjects (Fig. 6). CRP, PCSK9, ApoC-III, sICAM-1, AGT, FETUB and BGN were up-regulated in pathological human samples, presenting the same trend observed in ApoE^−/−^/LDLR^−/−^ plasma samples. In turn, PON-1 and VKDP-S were found down-regulated in FH sera, similar to the animal plasma samples, although variations in the concentration of the latter were minor. Further, a total of 24 metabolites were found significantly altered in human sera, highly similar to the trend observed in animal samples (Additional file 1: Table S8). Compared with the normolipidemic subjects, FH patients had remarkably higher Hcy, Ala as well as methylated Arg concentrations, but they also had lower levels of BCAAs and AAs engaged in NO generation. In contrast, among the dimethylated Args determined, the level of SDMA was substantially more elevated than ADMA in hyperlipidaemic subjects and ApoE^−/−^/LDLR^−/−^ plasma samples as compared to ApoE^−/−^/LDLR^−/−^ on atherogenic diets, where a massive increase in ADMA was observed that clearly illustrates that SDMA, rather than ADMA, might be a suitable marker of early atherosclerotic disease before the development of a lumen-compromising plaque. Gln and Tau, with potent anti-inflammatory/antioxidant properties, were also significantly down-regulated in FH sera, potentially suggesting the dysregulation of defences against oxidative stress. By contrast, MNA concentration was only slightly elevated in comparison to the strikingly high increase observed in ApoE^−/−^/LDLR^−/−^ plasma samples. Similarly, only a mild increase in Hyp (the elevated plasma level of which has been recognized as a possible risk factor associated with connective tissue injuries [37]) was identified in hyperlipidaemic patient samples vs normolipidemic samples. Most of these metabolomic alterations can be found in Fig. 7. Interestingly, the treatment of FH patients with cholesterol-lowering drugs (statins) normalized molecular disturbances induced by dyslipidaemia (Figs. 6, 7). Hence, to corroborate the discriminative potential of the molecules described above, further investigation was carried out on an additional cohort of cases (Additional file 1: Table S5). That study demonstrated a largely overlapped pattern of dysregulated metabolites/proteins when these two clinical cohorts were compared with each other (Additional file 1: Figures S4, S5, Table S9), thus confirming the value of a molecular signature that was ascribed to atherogenic dyslipidaemia.

**Fig. 8:**
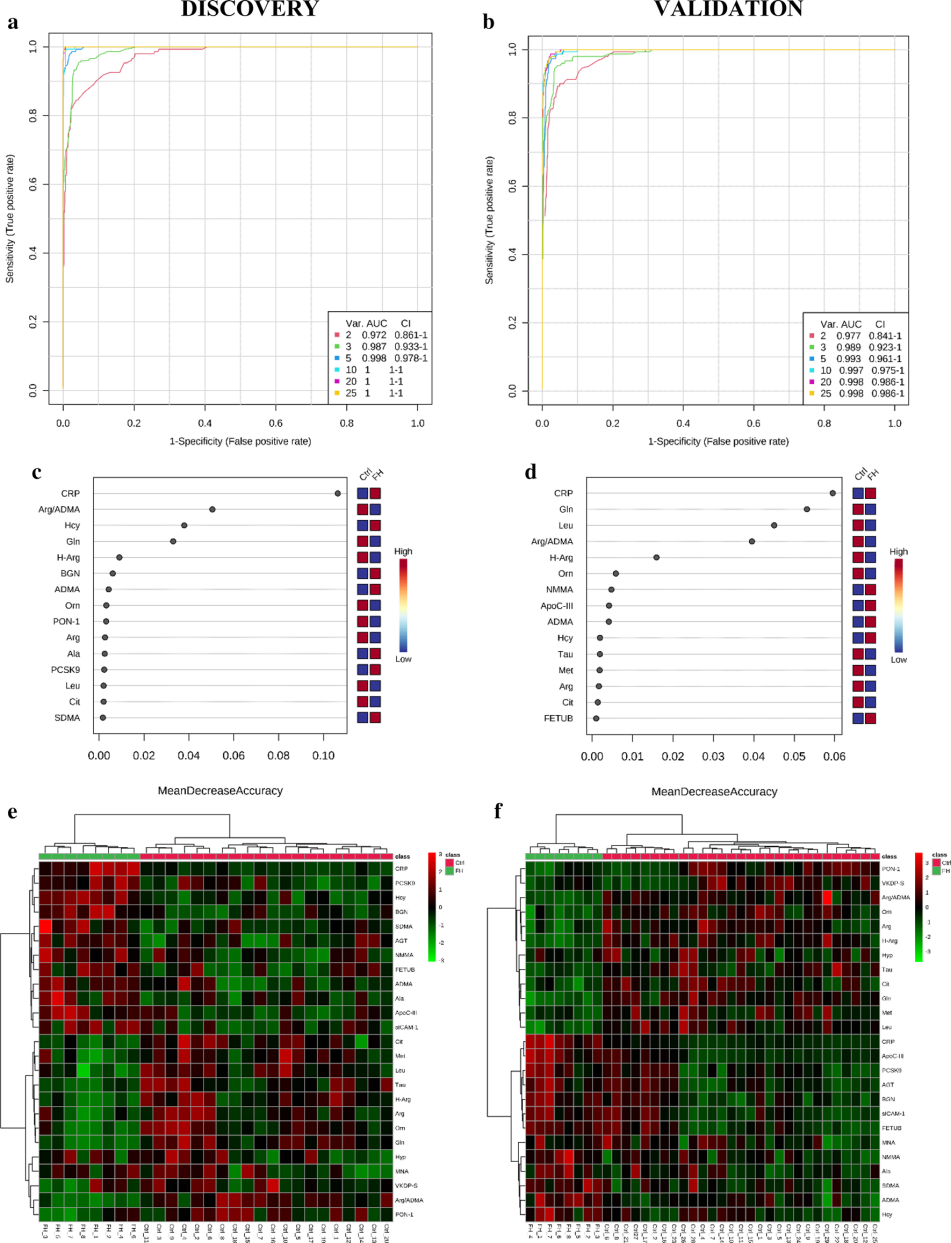
Receiver operating characteristic (ROC) curves (**a**–**d**), showing the ability of the potential biomarkers to distinguish between control subjects and FH patients, as well as heatmaps data visualization to depict variance across multiple variables (**e**, **f**). **a**, **b** Multivariate ROC curves of the six models in primary and validation cohorts have been generated employing random forest prediction model with combined features. We found that the model with 10 features entered showed excellent predictive performance with ROC AUC (area under the curve) values > 99% for both discovery and validation data. **c**, **d** 15 significant features were ranked based on their average importance (in group classification) during cross validation. **e**, **f** Color-coded maps that illustrate the relative abundance of feature combinations for two independent cohorts of cases

The essential proteins and metabolites that differentiated the two groups studied were further classified according to their most important biological functions in 9 groups: (1) inflammation and immune response (CRP, Gln), (2) lipid transport/metabolism (PCSK9, ApoC-III, Leu), (3) NO generation (Arg, Cit, Orn, H-Arg, NMMA, SDMA, ADMA, Met, Hcy), (4) adhesion of leukocytes to ECs/endothelial activation (sICAM-1), (5) blood coagulation/fibrinolysis (VKDP-S, MNA), (6) RAS over-activation (AGT), (7) detoxification of ROS (PON-1, Tau), (8) extracellular matrix reorganization/arterial remodelling (BGN, FETUB, Hyp) and (9) response to ischemia (Ala). Subsequently, the performance of these molecules in responding to atherogenic dyslipidaemia was evaluated by calculating the ROC curves of multiple features combined. The model that entered only 10 features (out of the 25 studied) exhibited area under the ROC curve value over 99% thus confirming its high predictive accuracy – the observation noted in the primary as well as validation cohorts (Fig. 8).

## Discussion

This study applied an integrated ‘–omic’ approach to characterize the main alterations, both at protein/peptide and metabolite levels, associated with dyslipidaemia and development of atherosclerosis. Although several similar approaches have been proposed to study the pathogenesis of the disease, they suffer from limited applicability to humans as these studies have been conducted only in pre-clinical settings, with a single ‘omics’ technology providing a limited view of the molecules dysregulated or on a limited number of specimens, making it difficult to draw general conclusions [22, 24–26]. To the best of our knowledge, this is the first time that such a large, comprehensive multi-omic approach has been employed for the study of atherosclerosis in both experimental and clinical (on two patient cohorts) settings, and thanks to this design, we hereby describe an original panel of proteins and metabolites corresponding to dyslipidaemia-induced atherosclerosis.

### Accumulation and retention of low-density lipoproteins (LDLs) in the arterial intima and their oxidative modification

According to the ‘response-to-retention’ hypothesis of atherogenesis, retention of apolipoprotein B/E-containing lipoproteins within the vessel wall is necessary for initiating an atherogenesis event [38]. In particular, proteoglycans with elongated glycosaminoglycan chains, including biglycan (BGN), perlecan and decorin seem to play a crucial role in this process [38, 39]. Positively charged structures on apolipoproteins can ionically interact with negatively charged sulphate and carboxylic acid groups on glycosaminoglycans, leading to prolonged retention of atherogenic lipoproteins in the subendothelial space. Furthermore, co-localization studies have demonstrated that in humans, BGN is a key proteoglycan mediating lipid retention, whereas in mice, both BGN and perlecan co-localize with apolipoproteins [40, 41]. This observation is consistent with our results showing BGN to be significantly up-regulated under pathological conditions, both in animals and humans. However, the particular role of BGN in atherosclerosis development has yet to be well defined; Tang et al. [42] have recently demonstrated that over-expression of BGN increased atherosclerosis, but BGN deficiency was not protective. On the other hand, increased vascular perlecan content in BGN-deficient mice (observed in these studies) might suggest a compensatory response of the vasculature for the BGN deficiency. Other potential effects of BGN, including its role in inflammation, regulation of growth factor availability and involvement in thrombin generation require further elucidation [43].

Interestingly, ApoC-III has been shown to increase the binding of LDLs to artery wall proteoglycans, although this increase was not related to its direct binding by itself [44]. In our studies, elevated plasma/serum ApoC-III concentration was one of the most characteristic features in pathologically changed samples and supports previous observations suggesting its critical role in promoting atherosclerosis and other metabolic diseases [45, 46]. Although ApoC-III atherogenicity was primarily attributed to hypertriglyceridemia [47] because of its ability to inhibit endothelial-bound lipoprotein lipase, recent evidence expands this function and reveals ApoC-III’s key role in endothelial activation and arterial inflammation [44]. Kawakami et al. [48] have demonstrated that ApoC-III (or ApoC-III-rich VLDL) can stimulate VCAM-1 and ICAM-1 expression in vascular ECs by activating PKC*β* and NF-κB, thus suggesting a novel mechanism for EC activation induced by dyslipidaemia. Its ability to induce endothelial dysfunction was further confirmed by the authors, supporting the view that ApoC-III dose-dependently attenuated insulin-stimulated eNOS activity without affecting its expression [44, 48]. Altogether, these findings clearly indicate that ApoC-III is a multifunctional protein that exerts pro-inflammatory effects on both monocytes and ECs and suggest that targeting ApoC-III may not only improve lipid metabolism but could also prevent the development of inflamed atherosclerotic plaques and their subsequent thrombotic complications.

In addition to the aforementioned proteins that are causally related to hyperlipidaemia and/or altered plasma lipid transport/metabolism, PCSK9 also impairs LDL-C clearance from the plasma, mostly by promoting LDL receptor degradation [49]. Here, we identified significant up-regulation of this protein in ApoE^−/−^/LDLR^−/−^ plasma samples as well as in untreated FH patients’ sera. Interestingly, high-dose statin therapy further increased PCSK9 levels in patients, highlighting that PCSK9 inhibitors might be a beneficial therapy for FH patients. Furthermore, recent studies suggested that PCSK9 could accelerate atherosclerosis through mechanisms beyond the degradation of the hepatic LDL receptor [50, 51]. Tang et al. [50] have demonstrated that PCSK9 gene interference could suppress atherosclerosis directly through decreasing vascular inflammation and inhibiting the TLR4/NF-κB signalling pathway without affecting plasma cholesterol levels in high-fat diet-fed ApoE-deficient mice. These findings strongly suggest that therapeutic PCSK9 inhibition may offer vascular benefits in addition to the reduction of plasma LDLs.

Lipoproteins retained in the arterial intima are exposed to oxidizing agents and enzymes that provide oxidized and aggregated LDL particles, which could be further recognized and internalized by macrophages [38]. This internalization is driven by the physical alteration of LDLs upon binding to proteoglycans, including changes in both ApoB/E conformation and lipid organization. The structural changes also make proteoglycan-bound LDLs more susceptible to oxidative modifications under conditions of decreased activity/expression of antioxidant enzymes and/or decreased concentrations of low-molecular-weight antioxidants. This increased susceptibility is consistent with our results showing that the expression of PON-1, catalase and GPx-3, as well as levels of several small-molecule antioxidants (Gln, Tau), decreased under pathological conditions accelerating LDL modification.

### Endothelial dysfunction and diminished NO bioavailability

EC dysfunction—a harbinger of multiple CVDs, as diverse as hypertension, atherosclerosis, coronary artery disease (CAD) and diabetes mellitus, was initially identified as impaired vasodilatation to specific stimuli; however, a broader understanding of this term includes not only impaired vasodilation but also a pro-inflammatory and pro-thrombotic state of endothelial phenotype [52, 53]. Our study shows a subset of deregulated metabolites (Arg and relatives) engaged in synthesis, release and/or activity of endothelium-derived NO, highlighting that eNOS activity and/or NO bioavailability was clearly diminished in the tested models. Hence, highly elevated levels of Hcy which reduces DDAH (dimethylarginine dimethylaminohydrolase) catabolic activity clearly manifest impaired this gaseous molecule availability [54]. Further, observed elevated plasma ApoC-III concentration seems to be a critical regulator of eNOS activity via preventing its phosphorylation by insulin-activated Akt, further leading to impaired NO generation by ECs that drives endothelial activation and enhances monocyte adherence to the vessel wall. It is not surprising, then, that areas of turbulent flow in the vessel wall, which have less endothelium-derived NO, are typically more prone to EC activation and atherosclerosis [55]. Indeed, our study supports previous observations that atherosclerotic lesions tend to develop in areas of vascular branching that are more exposed to turbulent rather than laminar flow [53, 55].

Interestingly, several papers reported that MNA—a terminal metabolite of nicotinamide clearance—at pharmacological doses, can improve endothelial function in humans and in experimental animals [13, 56, 57]. Domagala et al. [56] demonstrated that MNA ameliorated endothelial dysfunction measured as flow-mediated vasodilation in humans. In turn, Bar et al. [57] reported that MNA improved endothelial-dependent function in ApoE^−/−^/LDLR^−/−^ mice in vivo, while Mateuszuk et al. [13] found that MNA–induced anti-atherosclerotic effects in ApoE^−/−^/LDLR^−/−^ mice were linked to an improved NO–/PGI_2_–dependent function. In our study, a massive increase in plasma MNA was a dominant feature of the metabolite profile recognized in animals fed the LCHP diet. This observation was supported by other studies demonstrating that serum MNA was strongly associated with the presence and severity of human CAD [58]. In view of these results, we suggest that the increase in MNA concentration in atherosclerosis might have compensatory and vasoprotective nature [59].

### Endothelial inflammation

It is now widely accepted that atherosclerosis progression is driven by vascular inflammation (7) and the RAS, with its main effector—Ang II—has been recognized as one of the major mediators of vascular inflammation and atherogenesis [60]. Considerable evidence demonstrates that Ang II exerts its pro-inflammatory actions in the vascular wall by inducing the production of ROS, inflammatory cytokines (interleukin 6 (IL-6), monocyte chemoattractant protein-1 (MCP-1)) and adhesion molecules [60, 61]. Our studies highlighting RAS over-activation and, consequently, Ang II up-regulation both in an experimental model of atherosclerosis and the clinical setting support a modulatory role for the RAS in atherogenesis.

Presently, it is well known that Ang II influences all inflammatory response stages that include: vascular permeability, leukocyte recruitment, EC activation and vascular repair processes through mediators of cell growth and fibrosis [60]. Among the intracellular signalling pathways involved in Ang-II-induced vascular inflammation and remodelling, the production of ROS and the activation of pro-inflammatory transcription factors seem to play a critical role. In the vascular system, Ang II binds to its AT_1_ receptors inducing oxidative stress, mainly mediated by NAD(P)H oxidase over-expression/over-activation. In turn, increased ROS production leads to the uncoupling of eNOS, with a consequent decrease in NO bioavailability and activation of redox-sensitive pro-inflammatory transcription factors, such as NF-κB and Ets1, which trigger and/or potentiate the inflammatory cascade [60, 61]. Various studies have highlighted that IL-6, secreted peripherally at the site of vascular injury, is a principal inducer of acute-phase protein synthesis through hepatocyte stimulation [60, 62]. For instance, IL-6 stimulates AGT, FBG, CRP, SAA and several complement factors’ synthesis that propagate the downstream inflammatory response responsible for atherosclerosis. This observation is in alignment with the up-regulated levels of AGT, FBG and CRP/SAA that we have found in pathologically changed samples. Although the acute-phase reactants are thought to restore homeostasis mainly through promoting the clearance of infectious agents or by facilitating wound repair, they also exert significant lipid-metabolic and pro-inflammatory activities that, in turn, may exaggerate and sustain the process of atherogenesis [60]. Importantly, enhanced AGT delivery to the Ang II-processing enzymes present locally in the vasculature may further perpetuate the inflammatory cycle. Indeed, in our study, a positive correlation between circulating AGT concentrations and systemic/vascular Ang peptide concentrations (in ApoE^−/−^/LDLR^−/−^ mice) was noted. Moreover, it has recently been demonstrated that atherosclerotic vessels may be highly sensitive to locally produced Ang II as hypercholesterolemia leads to significant up-regulation of AT_1_ gene expression [63]. Altogether, our results support the existence of a biological positive-feedback loop, highlighting that vascular inflammation can be self-sustaining through the up-regulation of the vessel wall Ang II tone.

On the other hand, accumulating evidence suggests that CRP levels are one of the most powerful predictors of atherosclerosis and vascular death, offering prognostic value significantly exceeding that of LDL-C [61]. The mechanistic basis of this predictive value of CRP might be linked to its ability to decrease eNOS mRNA, augment ET-1 and up-regulate diverse adhesion molecules and chemoattractant chemokine expression. In addition, recent observations also suggest that CRP up-regulates NF-κB signalling in ECs, stimulates AT_1_R expression in vascular SMCs while attenuating endothelial progenitor cell (EPC) differentiation, survival, and angiogenic function, and this occurs, at least in part via an effect of CRP to suppress EPC eNOS expression [61, 64]. In our hand, elevated systemic CRP level was the most characteristic feature identified in FH patients’ sera.

As one of the features of endothelial inflammation is the stimulation of endothelial expression of cell-surface adhesion molecules (CAMs), such as VCAM-1, ICAM-1 and selectins that facilitate leukocyte trafficking in the vascular wall [61], we investigated the pattern of several soluble CAMs in patients with FH and ApoE/LDLR-deficient mice. A highly elevated plasma level of sVCAM-1, identified in ApoE^−/−^/LDLR^−/−^ mice, may serve as a marker of endothelial activation and local or systemic inflammation, whereas in humans, the level of sICAM-1 seems to have more prognostic value in regard to atherosclerosis or CVD development.

### Plaque formation, extracellular matrix reorganization and arterial remodelling

Once adherent, the monocytes transmigrate into the tunica intima, passing between the ECs, where they develop into macrophages and begin to express scavenger receptors, such as LOX-1, SR-A and CD36, which internalize modified lipoproteins [61]. The internalization process of these lipoprotein particles gives rise to lipid-laden macrophages or foam cells that characterize early atherosclerotic lesions. FBG, highly elevated in the plasma of our murine model, participates in atherosclerotic plaque formation mainly through the modulation of endothelial function and promotion of SMC proliferation and migration [65]. This glycoprotein has been recognized as an important component of the coagulation cascade and a critical determinant of blood viscosity and platelet aggregation. Although FBG and Hcy have both been reported as playing complementary roles in the platelet activation/aggregation cascade, that effect was attained by different mechanisms assigned to the factors investigated [66]. Furthermore, positive associations between plasma FBG concentrations and the risk of coronary heart disease and myocardial infarction (MI) have been found in several prospective epidemiological studies [65, 67]. However, it is still unclear whether these elevations in FBG levels are a causal factor in the development of atherosclerosis or, rather, an epiphenomenon of the atherogenic process. Our research seems to indicate that FBG functions as a pro-atherosclerotic factor, in addition to being a highly useful risk marker. Furthermore, consistent with the widely accepted assumption that coagulation and inflammation are not isolated elements because many proteins are involved in both processes, we have also found the down-regulated anticoagulant protein—VKDP-S—in FH sera that further accentuates a pro-thrombotic/hypercoagulable state in atherosclerosis.

During atherosclerosis progression, a decrease in the blood supply to the different tissues/organs may be taking place. Under conditions of ischemia, plasma/serum Ala appears to be increased, which may be due to myocardium release through the pyruvate transamination. Indeed, we have found significantly elevated levels of Ala in the sera of FH patients as well as in pathologically changed murine samples.

Interestingly, in the present study, FETUB was up-regulated in ApoE^−/−^/LDLR^−/−^ plasma samples, presenting a similar trend to that observed in human samples, although variations in the concentration in FH sera (as compared to controls) were minor. Jung et al. [68] have suggested that FETUB may be associated with atherosclerotic plaque vulnerability. They demonstrated a significantly increased FETUB expression in serum from patients with acute MI as compared to those with stable angina. This protein accelerated monocytes/macrophages migration and affected vascular plaque-stabilizing factors, including lipoprotein deposition and cytokine production in macrophages, MMP-2 activation in monocytes as well as the activation of apoptosis and MMP-2 in VSMCs [68, 69]. Moreover, in vivo administration of FETUB decreased the collagen accumulation and SMC mobilization and showed an increased number of macrophages in the vascular plaque [68]. Our research seems to add to the above observations, suggesting a critical role for this protein in the development of acute MI. Adding to these findings, a significantly higher plasma Hyp concentration was noted in the dyslipidaemic mice as compared to the wild types, albeit that the differences in human sera were minor. According to the above observations, FETUB and Hyp could be considered as novel predictors of a vulnerable plaque, as well as indicators of an increased inflammatory status that is generally associated with higher patient susceptibility for plaque rupture.

### Disruption of self-renewal capacity of the endothelium

A variety of evidence suggests that endothelial damage eventually represents a balance between the magnitude of injury and the capacity for repair [70]. Little is known regarding the mechanisms by which the vessel wall undergoes repair; however, there are many indications that circulating EPCs constitute one aspect of this repair process thereby limiting the formation of atherosclerotic lesions [71]. Moreover, ADMA has been recognized as an endogenous inhibitor of the mobilization, differentiation and function of EPCs [72], and Thum et al. [73] demonstrated that its high levels inversely correlated with low numbers and function of EPCs in patients with CAD. Our data seem to support the hypothesis that an impaired ability to restore vascular function might accelerate atherogenesis as an elevated ADMA level was one of the most distinctive features of the plasma metabolite profile of animals fed an LCHP diet as compared to other (AIN-93G or Western) diets. Interestingly, Foo et al. [74] also demonstrated that an LCHP diet (in ApoE^−/−^ mice) substantially reduced the number of bone marrow and peripheral blood EPCs, and EPCs from these mice (on the LCHP diet) manifested lower levels of activated (phosphorylated) Akt (kinase), important in its mobilization, proliferation and survival. Altogether, the above observations suggest that the development of novel drugs that effectively reduce ADMA levels would most likely lead to a novel therapeutic concept in the prevention and treatment of atherosclerosis (or CAD) based on the improved mobilization and function of EPCs, thus strengthening the vascular regenerative capacity.

## Limitations of the study

The authors are aware that extrapolating of the results gathered in mice models to human medicine has a number of limitations but rapid mice reproduction, ease of their genetic manipulation and rapid atherogenesis development in murine models are undisputable [75]. Here, we used ApoE/LDLR^−/−^ mice as they develop more severe hyperlipidaemia and atherosclerosis than single knockouts (ApoE^−/−^ or LDLR^−/−^, respectively) without a need of Western diet, and display a similar pattern of vascular inflammation and atherogenesis as humans [11, 15, 75]. Indeed, LDLR^−/−^ mice, compared to ApoE^−/−^ or ApoE/LDLR^−/−^, display modestly elevated plasma cholesterol levels and develop only mild atherosclerosis when fed a normal diet; however, they are highly responsive to high-fat/high cholesterol Western-type diets that affect the lipoprotein profile of these mice, amplifying atherosclerotic lesion development. Nonetheless, LDLR^−/−^ mice possess some advantages over ApoE^−/−^ mice in terms of comparison with human FH: (1) a more human-like lipid profile (as plasma cholesterol is carried more by LDL than in HDL particles but still not identical with typical human-like lipid pattern as reproduced in E3L.CETP mice [76]), (2) targeted inactivation of the LDL receptor does not impact inflammation as the deletion of ApoE, and (3) this model shares the characteristics observed in human FH (mutations in LDLR gene) [11, 75]. Although our study demonstrates a relative agreement between species in terms of multi-omic signature identified, some caution should be taken in extrapolating the results from animals to humans. More precisely, an estimated 70–95% of FH results from a heterozygous pathogenic variant in one of the following genes—LDLR, APOB, PCSK9—with the first one accounting for approximately 80% of FH [77]. Homozygous FH (HoFH) with pathogenic variants in one of these known genes (LDLR, APOB, PCSK9) affects a lower proportion of the population and is manifested by severe CAD (typically by the mid-20 s) and a high rate of either death or coronary bypass surgery. In contrast, in the current study, homozygous mice for the disrupted ApoE and LDLR genes were used, which obviously cannot reflect FH disease phenotype.

In the current work, the authors employed the classic targeted quantification assay using SRM mode with the aim of performing a precisely targeted measurement of dozens of metabolites in the matrix evaluated. However, we are aware that the utilization of this strategy in biological studies might be limited/not optimal due to its relatively low metabolite coverage and throughput capacity. Nevertheless, in modern metabolomics compromises must be reached when selecting among the features for analysis performance, including metabolite coverage, throughput, specificity, accuracy and feasibility for specific approaches, and adjustments in strategies must be attained depending on the analytical goals. AAs and their derivatives were selected as the subject of the study as abundant evidence indicated that they play a fundamental role in cardiovascular physiology and pathology [78]. Decades of research have established the importance of L-Arg, L-Gln, L-Trp and L-Cys in modulating vascular function via the formation of a myriad of metabolites, including a number of gases (nitric oxide, carbon monoxide, ammonia, hydrogen sulphide and sulphur dioxide) [78, 79]. The metabolism of AAs has been causally linked to the regulation of vascular homeostasis as well as immune cell function. Moreover, there is an emerging recognition that circulating levels of multiple AAs are altered in vascular-related disorders and that a more holistic approach that targets all of these AA derangements is required to restore circulatory function in blood vessels affected by lipid abnormalities [79]. Nonetheless, the recent advances in LC/MS instruments and analytical strategies have brought substantial progress in targeted metabolomics determinations, and new approaches in this field have enabled expanding the options for large-scale targeted metabolomics analyses that employ high-resolution instruments with parallel reaction monitoring (PRM) mode [80]. The strategies based on high-res/PRM techniques now constitute attractive modalities for quantitative metabolomics analysis and high-throughput biomarker discovery.

In addition, plasma samples have been used for -omics evaluations in experimental models, whereas in humans, serum was applied that could also have affected the quality or integrity of the data as well as the final conclusions. Albeit that several previous studies have clearly indicated that many target clinical parameters, such as metal ions and proteins/enzymes may possess different concentrations in the two different media, the composition of small molecules in plasma and serum is considered to be highly similar [81–83]. Some studies have also demonstrated that on a global level, metabolite association networks from plasma and serum fractions that were collected from the same individuals presented similar topologies, and only at a local level did some differences arise that were seen in higher concentrations of selected metabolites in serum than in plasma (which should be taken into account when analysing, comparing and interpreting the data) [84]. However, as we are more interested in the relationships among metabolite concentration levels rather than in their levels alone (at least in the context of our study), as long as the same blood preparation procedure is used, either matrix—plasma or serum—should generate similar results in (pre)clinical studies. On the other hand, it was also suggested that the lower protein content in serum might benefit metabolite analyses and improve the overall sensitivity, making serum more applicable for high-throughput biomarker profiling [81, 85]. Additionally, as different collection procedures and the coagulation cascade influence the fibrinogen and associated proteins [82], those were specifically excluded from the investigations of clinical samples to avoid sample-related biases in biomarker studies.

In recent years, high-throughput omics technologies, frequently defined as high-throughput biochemical assays, have provided an unprecedented opportunity to depict the biological system under investigation at multiple molecular levels [86–88]. However, the complexity of managing and integrating such multi-omics datasets continues to be a challenge, and the development of reliable strategies that can successfully tackle that complexity of multi-dimensional data is critical for gaining actionable knowledge in a precision medicine framework. Those recent data-driven methodologies/innovations (such as machine learning methodologies or network-based methods) have been developed/applied to respond to major challenges of stratified medicine, including patients’ phenotyping, biomarker discovery and drug repurposing; however, some constraints associated with the availability of the data for single omics individually, hosting options not dedicated for the multi-platform/multi-layered data, and the lack of relevant guidelines for quality and validation of data consistency should be improved before those tools might be deemed effective in a clinically meaningful way [86–88]. The authors are also aware of some limitations of the experimental design proposed as well as data accessibility in the current study. More specifically, the high-throughput sequencing technology was only applied in animal studies, thus providing limited insights into the complexity of biological processes. We also understand that only integrated approaches (based on complete multi-omics datasets) allow for comprehensive views of genetic, biochemical, proteomic and metabolic processes underlying the disease. However, considering an extrapolation of the approach selected to larger biological cohorts and multifaceted experimental designs in which a plethora of samples would need to be analysed in a realistic timeline, the feasibility of gathering clinically meaningful information was attained, and our study could be a starting point for more holistic investigations.

## Conclusions

Despite a number of limitations of this work, we identified 15 metabolites and 9 proteins as key molecular alterations in underlying atherogenic dyslipidaemia. Those metabolites/proteins were mainly related to lipid metabolism, LDL retention, oxidative stress, endothelial inflammation, hypercoagulation, arterial remodelling, activation of response to ischemia as well as impairment in the vascular regenerative capacity. A specific molecular panel, recognized here, could be proposed to monitor atherosclerosis development and progression to predict potential cardiovascular risk. However, as the vascular changes were not followed in patients that we studied, further analysis is necessary to confirm the relation of chosen biomarkers with the progression of clinical atherosclerosis. If these preliminary results are confirmed in a larger cohort of patients and its relation to clinical presentation in long-term analysis, they may be valuable for personalized prevention of dyslipidaemia-induced atherosclerosis.

## Supplementary information

**Additional file 1.** Laboratory data of animals, clinical characteristics of human subjects, ELISA results for animal samples, plasma and aortic angiotensin pattern in animals, effects of pro-atherogenic diets on the progression of atherosclerosis and survival of atherosclerotic mice, complete metabolomics dataset for animals and clinical cases, proteomic data for clinical validation cohort.

**Additional file 2.** The complete dataset involving results from untargeted (LC/MS) as well as targeted determinations (LC/MS or ELISA).

## Data Availability

All data generated or analysed during this study are included in this published article [and its additional files]. Any additional information that might help in replicating the procedures or reproducing the results will be available from the corresponding author on reasonable request.

## Abbreviations

AA(s): Amino acid(s)
ACE: Angiotensin-converting enzyme
ACN: Acetonitrile
ADMA: Asymmetric dimethylarginine
AGT: Angiotensinogen
AIN-93G: Control diet for laboratory rodents (formulation published by the American Institute of Nutrition)
Ala: Alanine
Ang: Angiotensin
ApoC-III: Apolipoprotein C-III
ApoE^−/−^/LDLR^−/−^: Gene-targeted, apolipoprotein E and LDL receptor-double knockout mice
Arg: Arginine
BGN: Biglycan
BMI: Body mass index
BCAA: Branched-chain AA
CAMs: Cell-surface adhesion molecules
CAD: Coronary artery disease
Cit: Citrulline
Cre: Creatinine
CRP: C-reactive protein
CVDs: Cardiovascular diseases
EC(s): Endothelial cell(s)
EPCs: Endothelial progenitor cells
ESI: Electrospray ionization
FBG: Fibrinogen
FETUB: Fetuin-B
FH: Familial hypercholesterolemia
Gln: Glutamine
GPx-3: Glutathione peroxidase 3
H-Arg: Homoarginine
HCD: Higher-energy collisional dissociation
Hcy: Homocysteine
HESI: Heated electrospray ionization probe
Hyl: 5-Hydroxylysine
Hyp: 4-Hydroxyproline
Ile: Isoleucine
IL-6: Interleukin 6
LCHP: Low-carbohydrate, high-Protein (diet)
LC/MS: Liquid chromatography/mass spectrometry
LDL-C: Low-density lipoprotein cholesterol
LDLs: Low-density lipoproteins
Leu: Leucine
MCP-1: Monocyte chemoattractant protein-1
Met: Methionine
MetPA: Metabolic pathway analysis
MI: Myocardial infarction
MNA: 1-Methylnicotinamide
MS: Mass spectrometry
NMMA: Monomethylarginine
nanoESI: Nanoelectrospray ion source
Orn: Ornithine
PON-1: Paraoxonase 1
PCSK9: Proprotein convertase subtilisin/kexin type 9
PSM: Peptide spectrum match
RAS: Renin–angiotensin system
ROC: Receiver operating characteristic (curves)
ROS: Reactive oxygen species
SAA: Serum amyloid A protein
SDMA: Symmetric dimethylarginine
sICAM-1: Soluble intercellular adhesion molecule-1
SRM: Selected reaction monitoring (mode)
sVCAM-1: Soluble vascular cell adhesion molecule-1
Tau: Taurine
VKDP-S: Vitamin K-dependent protein S
VSMCs: Vascular smooth muscle cells
WD: Western diet
WT: Wild-type mice
