# Supplementary material for: Multi-omic signatures of atherogenic dyslipidaemia: pre-clinical target identification and validation in humans
Source: J Transl Med. 2021 Jan 6;19:6. doi: 10.1186/s12967-020-02663-8 (PMC7789501; doi:10.1186/s12967-020-02663-8)
Supplement: Supplementary file 1 — Additional file 1. Laboratory data of animals, clinical characteristics of human subjects, ELISA results for animal samples, plasma and aortic angiotensin pattern in animals, effects of pro-atherogenic diets on the progression of atherosclerosis and survival of atherosclerotic mice, complete metabolomics dataset for animals and clinical cases, proteomic data for clinical validation cohort. [file 12967_2020_2663_MOESM1_ESM.docx]

**SUPPLEMENTARY MATERIAL**

**MULTI-OMIC SIGNATURES OF ATHEROGENIC DYSLIPIDAEMIA: PRE-CLINICAL TARGET IDENTIFICATION AND VALIDATION IN HUMANS**

Mariola Olkowicz^1,2*^, Izabela Czyzynska-Cichon^2^, Natalia Szupryczynska^3^, Renata B. Kostogrys^4^, Zdzislaw Kochan^3^, Janusz Debski^5^, Michal Dadlez^5^, Stefan Chlopicki^2,6^, Ryszard T. Smolenski^1*^

*^1^Department of Biochemistry, Faculty of Medicine, Medical University of Gdansk, 1 Debinki St., 80-211 Gdansk, Poland;*

*^2^Jagiellonian Centre for Experimental Therapeutics (JCET), Jagiellonian University, 14 Bobrzynskiego St., 30-348 Krakow, Poland;*

*^3^Department of Nutritional Biochemistry, Faculty of Health Sciences, Medical University of Gdansk, 7 Debinki St., 80-211 Gdansk, Poland;*

*^4^Department of Human Nutrition and Dietetics, Faculty of Food Technology, University of Agriculture in Krakow, 122 Balicka St., 30-149 Krakow, Poland*;

*^5^Mass Spectrometry Laboratory, Institute of Biochemistry and Biophysics, Polish Academy of Sciences, 5a Pawinskiego St., 02-106 Warsaw, Poland;*

*^6^Chair of Pharmacology, Jagiellonian University Medical College, 16 Grzegorzecka St., 31-531 Krakow, Poland*

* Correspondence: mariola.olkowicz@jcet.eu; rt.smolenski@gumed.edu.pl.

**Supplementary Table 1.** Characteristics and laboratory data of wild-type (C57BL/6) and ApoE^−/−^/LDLR^−/−^ mice.

|  | **C57BL/6**  **(n = 9)** | **ApoE^−/−^/LDLR^−/−^**  **(n = 7)** | **P-value** |
| --- | --- | --- | --- |
| BW [g] | 26.8 ± 2.4 | 27.7 ± 2.1 | 0.45 |
| HW [mg] | 104.5 ± 19.8 | 109.3 ± 18.3 | 0.63 |
| HW/BW×1,000 | 3.9 ± 0.6 | 3.9 ± 0.8 | 1.00 |
| T-Cho [mmol/L] | 1.87 ± 0.30 | 13.37 ± 0.48 | <0.0001 |
| TG [mmol/L] | 0.50 ± 0.12 | 2.82 ± 0.40 | <0.0001 |
| LDL-Cho [mmol/L] | 0.15 ± 0.03 | 7.15 ± 1.19 | <0.0001 |
| HDL-Cho [mmol/L] | 1.13 ± 0.18 | 0.93 ± 0.19 | 0.05 |
| FFA [mmol/L] | 1.13 ± 0.24 | 2.04 ± 0.34 | <0.0001 |
| Glucose [mg/dL] | 79.1 ± 6.5 | 81.6 ± 6.7 | 0.46 |

Values are expressed as mean ± SD; ApoE^−/−^/LDLR^−/−^, gene-targeted, apolipoprotein E and LDL receptor-double knockout mice; BW, body weight; HW, heart weight; T-Cho, total cholesterol; TG, triglycerides; LDL-Cho, low-density lipoprotein cholesterol; HDL-Cho, high-density lipoprotein cholesterol; FFA, free fatty acids.

**Supplementary Table 2.** Composition of experimental diets (%).

|  | **Control (AIN-93G)** | **WD** | **LCHP** |
| --- | --- | --- | --- |
| Corn starch  Casein  Sucrose  Soybean oil  Butter  Cellulose powder  Mineral mixture  Vitamin mixture  Choline | 53  20  10  7  –  5  4  1  0.25 | 15  20  34  –  21  5  4  1  0.25 | 5  52  12  –  21  5  4  1  0.25 |
| **Energy** | 3558 kcal/kg diet | 4432 kcal/kg diet | 4136 kcal/kg diet |

WD – Western diet; LCHP – Low-Carbohydrate, High-Protein diet.

**Supplementary Table 3.** Effect of experimental diets on body and organs’ weight and plasma biochemical markers in ApoE^−/−^/LDLR^−/−^ mice after 8 weeks of feeding. The data are presented as mean values ± SD or median with data range (minimum to maximum value). Statistical analyses were two-sided Student’s t-test or the non-parametric Mann-Whitney U test depending on the normality of distribution (Shapiro-Wilk test) and the variance homogeneity (Levene test).

|  | **Control (n = 6)** | **WD (n = 5)** | **P-value** | **LCHP (n = 5)** | **P-value** |
| --- | --- | --- | --- | --- | --- |
| Initial BW [g] | 21.6 (20.3–21.9) | 22.3 (18.8–25.2) | 0.378 | 22.1 (18.4–22.2) | 0.411 |
| Final BW [g] | 21.7 (19.3–23.7) | 23.1 (20.9–25.9) | 0.230 | 22.4 (19.3–23.2) | 0.648 |
| Liver weight [g/100 g BW] | 5.00 (4.70–5.32) | 5.86 (5.36–6.41)* | 0.005 | 6.20 (5.52–7.91)* | 0.008 |
| Kidney weight [g/100 g BW] | 1.15 ± 0.07 | 1.25 ± 0.07* | 0.015 | 1.49 ± 0.15* | 0.008 |
| Glucose [mg/dL] | 112.5 (93.0–123.0) | 122.0 (92.0–130.0) | 0.337 | 112.0 (110.0–122.0) | 1.000 |
| T-Cho [mmol/L] | 22.0 ± 2.9 | 43.3 ± 4.8* | <0.0001 | 48.0 ± 3.7* | <0.0001 |
| LDL-Cho [mmol/L] | 9.14 (6.96–10.83) | 20.5 (16.2–23.5)* | 0.005 | 22.5 (19.4–26.8)* | 0.008 |
| HDL-Cho [mmol/L] | 0.83 (0.69–0.96) | 0.63 (0.63–0.78)* | 0.013 | 0.72 (0.51–0.93) | 0.270 |
| TG [mmol/L] | 2.59 ± 0.90 | 1.84 ± 0.57 | 0.148 | 3.80 ± 0.85 | 0.069 |
| ALT [U/L] | 57.4 ± 22.0 | 83.4 ± 39.9 | 0.230 | 73.7 ± 74.7 | 0.655 |
| AST [U/L] | 156.1 ± 60.4 | 792.1 ± 169.3* | <0.0001 | 90.0 ± 35.6 | 0.083 |

**P* < 0.05 vs AIN-93G (control diet); BW, body weight; ALT, alanine aminotransferase; AST, aspartate aminotransferase.

**Supplementary Table 4.** Demographic and clinical characteristics of FH cases and control subjects. Values are presented as mean ± SD or number (discovery cohort).

| **Variables** | **Control (n = 20)** | **FH patients**  **without statin treatment**  **(n = 8)** | **FH patients receiving statin therapy**  **(n = 12)** |
| --- | --- | --- | --- |
| Age (years)  Gender (male/female, *n*)  BMI (kg/m^2^) | 43 ± 9  5/15  25.0 ± 3.2 | 37 ± 14  2/6  22.7 ± 4.4 | 46 ± 16  3/9  26.3 ± 2.6 |
| *Biochemical Data* |  |  |  |
| Total cholesterol (mg/dL)  LDL-cholesterol (mg/dL)  HDL-cholesterol (mg/dL)  Triglycerides (mg/dL) | 223.0 ± 38.7  135.8 ± 21.5  62.0 ± 10.8  73.3 ± 21.1 | 320.8 ± 93.5**  176.2 ± 50.3*  49.0 ± 18.5*  171.8 ± 162.0* | 258.8 ± 88.6  162.9 ± 51.5  67.2 ± 9.8##  124.9 ± 73.7 |
| *Comorbidity (n)* |  |  |  |
| Hypertension  Diabetes mellitus  Chronic kidney disease  Coronary artery disease | 2  0  0  0 | 1  0  0  0 | 1  1  0  0 |
| *Medication (n)* |  |  |  |
| Statins  Anticoagulant and antiplatelet drugs  ACEIs/ARBs/MRAs  β-Blockers  Calcium channel antagonists  Diuretics  Anti-diabetic drugs | 0  0  1  1  0  1  1 | 0  0  0  1  0  0  0 | 12  0  0  0  1  0  0 |

BMI, body mass index; LDL, low-density lipoprotein; HDL, high-density lipoprotein; ACEIs, angiotensin-converting enzyme inhibitors; ARBs, angiotensin II type I receptor blockers; MRAs, mineralocorticoid receptor antagonists. **P* < 0.05, ***P* < 0.01 vs Controls; ##*P* < 0.01 vs FH patients not treated with statins. For comparison of independent groups one-way ANOVA with Bonferroni’s post-hoc test was used.

**Supplementary Table 5.** Demographic and clinical characteristics of FH cases and control subjects. Values are presented as mean ± SD or number (validation cohort).

| **Variables** | **Control (n = 29)** | **FH patients**  **without statin treatment**  **(n = 8)** | **FH patients receiving statin therapy**  **(n = 21)** |
| --- | --- | --- | --- |
| Age (years)  Gender (male/female, *n*)  BMI (kg/m^2^) | 42 ± 14  7/22  24.7 ± 3.8 | 42 ± 17  0/8  23.1 ± 4.0 | 46 ± 14  3/18  26.0 ± 4.9 |
| *Biochemical Data* |  |  |  |
| Total cholesterol (mg/dL)  LDL-cholesterol (mg/dL)  HDL-cholesterol (mg/dL)  Triglycerides (mg/dL) | 225.3 ± 45.2  132.5 ± 24.6  60.0 ± 15.1  101.4 ± 31.5 | 278.1 ± 45.5*  173.0 ± 49.5**  67.1 ± 12.2  114.1 ± 39.6 | 250.9 ± 56.3  151.0 ± 30.7  63.5 ± 9.8  108.0 ± 56.3 |
| *Comorbidity (n)* |  |  |  |
| Hypertension  Diabetes mellitus  Chronic kidney disease  Coronary artery disease | 1  1  0  0 | 0  0  0  0 | 3  0  1  0 |
| *Medication (n)* |  |  |  |
| Statins  Anticoagulant and antiplatelet drugs  ACEIs/ARBs/MRAs  β-Blockers  Calcium channel antagonists  Diuretics  Anti-diabetic drugs | 1  0  1  1  1  1  2 | 0  0  0  0  0  0  0 | 21  0  1  3  1  0  0 |

BMI, body mass index; LDL, low-density lipoprotein; HDL, high-density lipoprotein; ACEIs, angiotensin-converting enzyme inhibitors; ARBs, angiotensin II type I receptor blockers; MRAs, mineralocorticoid receptor antagonists. **P* < 0.05, ***P* < 0.01 vs Controls. For comparison of independent groups one-way ANOVA with Bonferroni’s post-hoc test was used.

**Supplementary Table 6.** Plasma amino acids and related metabolites concentrations (µM) in wild-type (C57BL/6) and ApoE^−/−^/LDLR^−/−^ mice. Data are reported as mean ± SEM.

|  | **C57BL/6**  **(n = 9)** | **ApoE^−/−^/LDLR^−/−^**  **(n = 7)** | **Difference**  **(%)** | **P-value** |
| --- | --- | --- | --- | --- |
| Taurine  4-Hydroxyproline  Asparagine  Aspartic Acid  Serine  Glycine  Betaine  Sarcosine  Glutamic Acid  Glutamine  Threonine  Alanine  Citrulline  Proline  β-Alanine  Cystine  α-Amino-n-butyric Acid  γ-Aminobutyric Acid  1-Methylnicotinamide  Creatinine  β-Aminoisobutyric Acid  Cystathionine  Valine  Methionine  Homocysteine  Tyrosine  3-Methylhistidine  Histidine  Ornithine  5-Hydroxylysine  1-Methylhistidine  Isoleucine  Leucine  Lysine  Argininosuccinic Acid  Arginine  Phenylalanine  Monomethylarginine  Asym(Me)_2_Arg  Sym(Me)_2_Arg  Homoarginine  Anserine  Carnosine  Tryptophan | 122.7 ± 9.0  5.75 ± 0.22  33.0 ± 3.6  11.5 ± 1.8  165.7 ± 7.3  324.8 ± 34.5  49.4 ± 3.0  7.61 ± 0.67  88.4 ± 13.1  469.8 ± 49.5  162.8 ± 17.2  388.3 ± 16.4  55.7 ± 2.5  66.1 ± 2.9  7.72 ± 1.06  7.54 ± 0.86  20.2 ± 1.9  0.95 ± 0.29  0.44 ± 0.03  15.9 ± 0.9  4.38 ± 0.48  0.87 ± 0.10  177.2 ± 13.1  36.0 ± 1.9  8.05 ± 0.52  45.2 ± 1.7  2.10 ± 0.17  48.9 ± 3.9  42.6 ± 3.5  4.00 ± 0.21  5.11 ± 0.31  128.2 ± 5.3  142.4 ± 8.6  131.1 ± 7.8  0.32 ± 0.04  53.1 ± 3.5  91.4 ± 5.1  0.055 ± 0.006  0.48 ± 0.05  0.13 ± 0.02  1.99 ± 0.15  1.81 ± 0.22  1.57 ± 0.19  81.3 ± 3.8 | 83.7 ± 9.2  8.49 ± 0.44  29.3 ± 3.4  12.5 ± 2.2  173.3 ± 16.1  358.4 ± 32.4  49.6 ± 2.4  8.98 ± 0.81  97.5 ± 8.5  327.9 ± 19.4  151.2 ± 12.2  471.8 ± 25.8  44.5 ± 4.3  69.7 ± 4.0  7.01 ± 1.53  4.22 ± 1.09  18.5 ± 1.0  0.80 ± 0.22  0.83 ± 0.07  19.9 ± 1.1  6.24 ± 1.13  0.97 ± 0.13  155.3 ± 12.9  29.5 ± 2.3  11.3 ± 0.6  38.1 ± 2.9  2.44 ± 0.29  57.9 ± 5.7  32.6 ± 2.6  6.37 ± 0.68  3.58 ± 0.33  88.5 ± 8.1  89.2 ± 7.2  121.4 ± 10.4  0.40 ± 0.09  41.4 ± 3.8  77.2 ± 6.7  0.072 ± 0.002  0.65 ± 0.04  0.21 ± 0.03  1.39 ± 0.07  0.84 ± 0.07  0.95 ± 0.10  51.1 ± 1.9 | –31.8%  +47.7%  NS  NS  NS  NS  NS  NS  NS  –30.2%  NS  +21.5%  –20.1%  NS  NS  –44.0%  NS  NS  +88.6%  +25.2%  NS  NS  NS  –18.1%  +39.9%  –15.7%  NS  NS  –23.5%  +59.3%  –29.9%  –31.0%  –37.4%  NS  NS  –22.0%  NS  +30.9%  +35.4%  +61.5%  –30.2%  –53.6%  –39.5%  –37.1% | <0.010  <0.001  0.478  0.728  0.649  0.500  0.961  0.210  0.595  0.031  0.612  0.013  0.032  0.467  0.700  0.029  0.480  0.701  <0.001  0.013  0.122  0.545  0.262  0.045  0.001  0.043  0.305  0.199  0.047  0.002  0.005  <0.001  <0.001  0.459  0.394  0.041  0.108  0.031  0.024  0.037  0.005  0.002  0.019  <0.001 |
| **AA Ratios** |  |  |  |  |
| Arg/ADMA ratio  Orn/Arg ratio  Fischer’s ratio* | 119.6 ± 12.1  0.84 ± 0.05  3.29 ± 0.09 | 64.5 ± 6.7  0.81 ± 0.07  2.94 ± 0.19 | –46.1%  NS  NS | 0.003  0.725  0.095 |

* Fischer's ratio – branched-chain amino acids (BCAAs): valine, isoleucine, leucine/aromatic amino acids (AAAs): phenylalanine, tyrosine.

**Supplementary Table 7.** Plasma amino acids and related metabolites concentrations (µM) in 6-month-old ApoE^−/−^/LDLR^−/−^ mice fed for 2 months: Control-AIN-93G (*n* = 6), Western (WD, *n* = 5) and Low-Carbohydrate, High-Protein (LCHP, *n* = 5) diet, respectively. Data are reported as mean ± SEM.

|  | **AIN-93G**  **(n = 6)** | **Western**  **(n = 5)** | **LCHP**  **(n = 5)** |
| --- | --- | --- | --- |
| Taurine  4-Hydroxyproline  Asparagine  Aspartic Acid  Serine  Glycine  Betaine  Sarcosine  Glutamic Acid  Glutamine  Threonine  Alanine  Citrulline  Proline  β-Alanine  Cystine  α-Amino-n-butyric Acid  γ-Aminobutyric Acid  1-Methylnicotinamide  Creatinine  β-Aminoisobutyric Acid  Cystathionine  Valine  Methionine  Homocysteine  Tyrosine  3-Methylhistidine  Histidine  Ornithine  5-Hydroxylysine  1-Methylhistidine  Isoleucine  Leucine  Lysine  Argininosuccinic Acid  Arginine  Phenylalanine  Monomethylarginine  Asym(Me)_2_Arg  Sym(Me)_2_Arg  Homoarginine  Anserine  Carnosine  Tryptophan | 105.2 ± 3.6  5.20 ± 0.22  39.3 ± 3.1  21.4 ± 2.4  175.9 ± 4.1  373.1 ± 6.4  99.3 ± 3.4  6.15 ± 0.31  80.7 ± 3.7  453.7 ± 20.2  135.4 ± 4.4  469.7 ± 17.6  63.3 ± 3.4  116.9 ± 7.4  10.8 ± 0.5  10.5 ± 0.5  13.4 ± 0.6  1.08 ± 0.09  0.91 ± 0.02  27.3 ± 0.9  7.11 ± 0.29  1.03 ± 0.04  172.0 ± 4.1  45.4 ± 2.7  10.8 ± 0.4  74.8 ± 4.2  1.44 ± 0.04  69.9 ± 2.2  42.7 ± 4.0  4.46 ± 0.26  2.29 ± 0.06  97.6 ± 4.1  101.7 ± 5.1  173.4 ± 4.0  0.70 ± 0.05  75.9 ± 3.3  76.4 ± 4.0  0.075 ± 0.005  0.64 ± 0.02  0.20 ± 0.01  2.06 ± 0.09  5.73 ± 0.23  2.76 ± 0.13  82.6 ± 3.8 | 127.3 ± 4.6  7.87 ± 0.57**  33.7 ± 3.0  30.3 ± 2.3*  135.5 ± 4.9***  305.7 ± 5.8***  86.1 ± 2.2*  11.0 ± 0.6***  101.4 ± 5.4*  446.7 ± 23.4  137.5 ± 5.5  568.7 ± 15.0**  52.4 ± 2.2  73.4 ± 2.2***  7.26 ± 0.42***  8.59 ± 0.59  25.3 ± 0.8***  1.72 ± 0.19**  1.19 ± 0.04  17.7 ± 1.0***  10.8 ± 0.6***  1.09 ± 0.07  136.0 ± 3.9**  32.0 ± 2.2**  12.1 ± 0.5  48.1 ± 3.9***  1.94 ± 0.06***  58.5 ± 3.7  22.8 ± 1.2***  7.09 ± 0.24***  2.17 ± 0.06  127.2 ± 3.8**  37.1 ± 2.3***  105.4 ± 4.5***  0.15 ± 0.01***  47.1 ± 5.6***  72.7 ± 4.0  0.081 ± 0.005  0.86 ± 0.04**  0.22 ± 0.01  1.87 ± 0.12  4.88 ± 0.28  1.66 ± 0.08***  77.0 ± 3.3 | 66.6 ± 2.9*##  7.00 ± 0.44*  38.6 ± 2.4  14.5 ± 1.0###  131.6 ± 3.4***  205.8 ± 10.1***###  85.7 ± 2.8*  8.75 ± 0.59**#  135.3 ± 6.5***##  218.3 ± 17.5***###  205.4 ± 8.3***###  558.5 ± 11.4**  52.3 ± 2.9  115.4 ± 5.5###  4.77 ± 0.36***##  6.76 ± 0.57***  17.7 ± 0.8**###  1.43 ± 0.02  3.43 ± 0.14***###  14.4 ± 0.5***  12.1 ± 0.4***  0.51 ± 0.03***###  184.3 ± 9.2###  35.0 ± 1.9*  13.1 ± 0.4**  77.3 ± 2.6###  1.59 ± 0.07##  89.0 ± 4.0**###  31.1 ± 2.1*  7.82 ± 0.33***  1.78 ± 0.05***##  262.2 ± 6.5***###  111.5 ± 4.4###  170.1 ± 7.4###  1.25 ± 0.07***###  51.3 ± 3.3***  120.1 ± 4.4***###  0.087 ± 0.003  1.52 ± 0.06***###  0.41 ± 0.01***###  1.74 ± 0.05  3.49 ± 0.22***##  1.54 ± 0.09***  60.7 ± 2.1**# |
| **AA Ratios** |  |  |  |
| Arg/ADMA ratio  Orn/Arg ratio  Fischer’s ratio^ | 117.3 ± 4.9  0.56 ± 0.04  2.47 ± 0.09 | 54.1 ± 4.5***  0.51 ± 0.07  2.51 ± 0.15 | 34.0 ± 3.4***#  0.62 ± 0.06  2.83 ± 0.07 |

^ BCAAs/AAAs ratio (branched-chain amino acids/aromatic amino acids); **P* < 0.05, ***P* < 0.01, ****P* < 0.001 vs AIN-93G (control diet); #*P* < 0.05, ##*P* < 0.01, ###*P* < 0.001 vs Western diet.

**Supplementary Table 8.** Plasma amino acids and related metabolites concentrations (µM) of FH cases and control subjects. Data are presented as mean ± SEM (discovery cohort).

|  | **Controls**  **(n = 20)** | **FH patients**  **without statin treatment**  **(n = 8)** | **FH patients receiving statin therapy**  **(n = 12)** |
| --- | --- | --- | --- |
| Taurine  4-Hydroxyproline  Asparagine  Aspartic Acid  Serine  Glycine  Betaine  Sarcosine  Glutamic Acid  Glutamine  Threonine  Alanine  Citrulline  Proline  β-Alanine  Cystine  α-Amino-n-butyric Acid  γ-Aminobutyric Acid  1-Methylnicotinamide  Creatinine  β-Aminoisobutyric Acid  Cystathionine  Valine  Methionine  Homocysteine  Tyrosine  3-Methylhistidine  Histidine  Ornithine  5-Hydroxylysine  1-Methylhistidine  Isoleucine  Leucine  Lysine  Argininosuccinic Acid  Arginine  Phenylalanine  Monomethylarginine  Asym(Me)_2_Arg  Sym(Me)_2_Arg  Homoarginine  Anserine  Carnosine  Tryptophan | 68.8 ± 5.9  8.66 ± 0.79  40.5 ± 3.0  11.3 ± 0.8  105.0 ± 6.3  251.3 ± 23.8  90.8 ± 5.8  5.81 ± 0.56  103.6 ± 5.9  533.6 ± 20.6  225.0 ± 16.5  332.0 ± 19.6  34.5 ± 2.2  220.9 ± 18.4  10.8 ± 2.1  10.0 ± 1.3  27.1 ± 1.7  0.66 ± 0.13  0.23 ± 0.03  77.9 ± 4.7  3.55 ± 0.54  0.14 ± 0.04  260.5 ± 13.8  22.7 ± 1.2  9.4 ± 0.5  91.3 ± 4.4  4.34 ± 0.89  152.4 ± 6.6  63.4 ± 4.4  2.68 ± 0.24  5.60 ± 0.52  131.4 ± 6.8  162.5 ± 6.4  130.9 ± 5.7  0.080 ± 0.009  90.6 ± 5.2  86.3 ± 3.4  0.047 ± 0.004  0.33 ± 0.03  0.28 ± 0.02  2.22 ± 0.16  0.033 ± 0.007  0.12 ± 0.01  68.2 ± 2.6 | 35.2 ± 3.4**  9.40 ± 1.17  35.6 ± 5.3  8.38 ± 1.26  74.7 ± 8.6*  200.7 ± 25.8  66.2 ± 6.1*  5.79 ± 0.63  72.8 ± 5.0**  337.9 ± 15.9***  178.5 ± 20.8  480.4 ± 50.3*  21.7 ± 1.7**  183.4 ± 33.3  9.20 ± 1.70  7.59 ± 1.71  21.0 ± 2.0  0.50 ± 0.17  0.29 ± 0.03  74.3 ± 9.0  4.89 ± 0.76  0.12 ± 0.07  166.8 ± 16.4***  16.0 ± 2.3*  15.8 ± 1.5***  45.9 ± 3.7***  5.53 ± 1.40  111.3 ± 9.7*  32.6 ± 5.5***  2.98 ± 0.24  4.06 ± 0.51  77.3 ± 7.9***  115.0 ± 10.8**  82.1 ± 11.0***  0.053 ± 0.012  66.2 ± 7.8*  65.0 ± 4.0**  0.069 ± 0.006*  0.48 ± 0.03*  0.46 ± 0.06**  1.02 ± 0.14***  0.028 ± 0.009  0.05 ± 0.01**  44.9 ± 3.8*** | 38.7 ± 6.7**  10.3 ± 1.6  32.7 ± 2.9  9.28 ± 0.87  71.0 ± 7.4**  187.7 ± 25.8  76.8 ± 6.3  5.54 ± 0.52  89.2 ± 6.4  336.4 ± 19.9***  211.2 ± 17.6  464.5 ± 37.8**  26.0 ± 2.7*  177.0 ± 16.6  9.31 ± 1.39  5.79 ± 1.33  23.4 ± 1.8  0.63 ± 0.12  0.26 ± 0.03  75.3 ± 4.2  4.55 ± 0.60  0.08 ± 0.01  211.5 ± 11.1*  16.7 ± 1.3*  12.9 ± 1.1*  53.1 ± 4.6***  6.55 ± 1.46  112.5 ± 11.4**  44.1 ± 4.2*  3.15 ± 0.39  4.78 ± 0.37  99.9 ± 9.6*  137.7 ± 8.7  90.1 ± 6.4***  0.057 ± 0.009  75.2 ± 6.6  71.0 ± 4.7*  0.062 ± 0.006  0.43 ± 0.05  0.38 ± 0.04  1.28 ± 0.12***  0.018 ± 0.005  0.05 ± 0.02**  41.8 ± 2.3*** |
| **AA Ratios** |  |  |  |
| Arg/ADMA ratio  Orn/Arg ratio  Fischer’s ratio^ | 303.3 ± 17.1  0.71 ± 0.04  3.14 ± 0.09 | 136.5 ± 11.5***  0.49 ± 0.04**  3.25 ± 0.23 | 204.0 ± 28.5**  0.60 ± 0.05  3.69 ± 0.15* |

^ BCAAs/AAAs ratio (branched-chain amino acids/aromatic amino acids); **P* < 0.05, ***P* < 0.01, ****P* < 0.001 vs Controls. For comparison of independent groups one-way ANOVA with Bonferroni’s post-hoc test was performed.

**Supplementary Table 9.** Plasma amino acids and related metabolites concentrations (µM) of FH cases and control subjects. Data are presented as mean ± SEM (validation cohort).

|  | **Controls**  **(n = 29)** | **FH patients**  **without statin treatment**  **(n = 8)** | **FH patients receiving statin therapy**  **(n = 21)** |
| --- | --- | --- | --- |
| Taurine  4-Hydroxyproline  Asparagine  Aspartic Acid  Serine  Glycine  Betaine  Sarcosine  Glutamic Acid  Glutamine  Threonine  Alanine  Citrulline  Proline  β-Alanine  Cystine  α-Amino-n-butyric Acid  γ-Aminobutyric Acid  1-Methylnicotinamide  Creatinine  β-Aminoisobutyric Acid  Cystathionine  Valine  Methionine  Homocysteine  Tyrosine  3-Methylhistidine  Histidine  Ornithine  5-Hydroxylysine  1-Methylhistidine  Isoleucine  Leucine  Lysine  Argininosuccinic Acid  Arginine  Phenylalanine  Monomethylarginine  Asym(Me)_2_Arg  Sym(Me)_2_Arg  Homoarginine  Anserine  Carnosine  Tryptophan | 90.6 ± 4.5  8.77 ± 0.60  66.4 ± 2.9  10.86 ± 0.52  85.0 ± 3.8  208.1 ± 16.3  84.1 ± 3.3  6.24 ± 0.34  78.5 ± 3.1  636.3 ± 19.9  186.9 ± 10.0  301.3 ± 18.5  41.2 ± 2.0  192.1 ± 10.0  8.71 ± 0.63  8.20 ± 1.26  22.7 ± 0.9  0.57 ± 0.06  0.33 ± 0.02  91.0 ± 3.5  3.14 ± 0.32  0.12 ± 0.02  252.8 ± 11.4  19.8 ± 1.2  10.3 ± 0.5  102.3 ± 5.0  4.24 ± 0.48  148.3 ± 5.2  54.6 ± 2.4  2.19 ± 0.15  5.46 ± 0.40  131.9 ± 3.7  171.4 ± 4.8  107.5 ± 4.3  0.070 ± 0.007  78.4 ± 3.1  102.9 ± 3.2  0.050 ± 0.002  0.40 ± 0.02  0.29 ± 0.02  1.88 ± 0.06  0.034 ± 0.006  0.31 ± 0.04  68.6 ± 2.3 | 60.9 ± 2.7**  7.91 ± 0.72  57.5 ± 5.0  9.43 ± 1.08  73.7 ± 7.1  190.5 ± 32.1  75.5 ± 7.3  5.37 ± 0.40  63.7 ± 2.4*  370.1 ± 22.2***  147.9 ± 9.1  420.5 ± 47.8*  27.6 ± 2.5**  152.7 ± 14.4  10.12 ± 1.75  4.67 ± 0.80  21.8 ± 1.3  0.51 ± 0.11  0.38 ± 0.04  86.9 ± 6.8  2.77 ± 0.64  0.12 ± 0.03  174.8 ± 10.7**  13.1 ± 1.0**  14.7 ± 1.4**  73.5 ± 7.0*  5.42 ± 0.81  106.3 ± 5.5***  33.0 ± 3.4***  1.98 ± 0.18  4.49 ± 0.54  82.9 ± 4.5***  118.4 ± 6.4***  88.8 ± 4.7  0.062 ± 0.014  54.2 ± 4.3***  78.4 ± 5.7***  0.067 ± 0.005*  0.58 ± 0.04***  0.41 ± 0.03**  1.21 ± 0.05***  0.028 ± 0.007  0.25 ± 0.04  64.9 ± 4.7 | 77.9 ± 5.9  10.49 ± 0.93  68.5 ± 4.1  9.10 ± 0.60  86.4 ± 4.7  216.5 ± 13.3  83.3 ± 5.5  6.41 ± 0.36  58.8 ± 3.0***  404.0 ± 11.8***  189.0 ± 11.0  375.8 ± 18.7*  30.9 ± 1.8***  219.8 ± 15.3#  7.48 ± 0.95  8.06 ± 1.10  20.1 ± 1.0  0.56 ± 0.09  0.35 ± 0.03  80.4 ± 4.4  3.10 ± 0.40  0.14 ± 0.02  155.9 ± 8.7***  14.7 ± 0.7**  13.7 ± 0.8**  87.3 ± 4.7  5.49 ± 0.44  129.0 ± 5.3*  38.0 ± 2.7***  2.62 ± 0.23  4.83 ± 0.41  84.4 ± 2.6***  126.6 ± 3.9***  108.4 ± 5.4  0.069 ± 0.009  62.4 ± 2.4***  71.9 ± 2.9***  0.061 ± 0.005  0.50 ± 0.03**  0.35 ± 0.03  1.53 ± 0.04***#  0.036 ± 0.006  0.29 ± 0.04  60.7 ± 2.7 |
| **AA Ratios** |  |  |  |
| Arg/ADMA ratio  Orn/Arg ratio  Fischer’s ratio^ | 209.1 ± 13.2  0.72 ± 0.03  2.75 ± 0.07 | 94.9 ± 5.9***  0.61 ± 0.05  2.53 ± 0.13 | 130.8 ± 7.5***  0.62 ± 0.04  2.36 ± 0.10** |

^ BCAAs/AAAs ratio (branched-chain amino acids/aromatic amino acids); **P* < 0.05, ***P* < 0.01, ****P* < 0.001 vs Controls; #*P* < 0.05 vs FH. For comparison of independent groups one-way ANOVA with Bonferroni’s post-hoc test was performed.

**Supplementary Fig. 1.** *Immunoassay-based verification of selected candidate proteins in plasma samples of the tested animals.* ELISA was used to measure plasma levels of serum amyloid A (SAA), proprotein convertase subtilisin/kexin type 9 (PCSK9), soluble vascular cell adhesion molecule-1 (sVCAM-1), fibrinogen (FBG), angiotensinogen (AGT), and glutathione peroxidase 3 (GPx-3) in ApoE^−/−^/LDLR^−/−^ (*n* = 6) and WT (*n* = 6) mice. Data represent mean ± SEM. **P* < 0.05, ***P* < 0.01, ****P* < 0.001 vs WT.

**A**

**B**

**Supplementary Fig. 2.** *Renin-angiotensin system alterations in ApoE^−/−^/LDLR^−/−^ mice.* Plasma (**A**) and aortic (**B**) angiotensin profile in 7-month-old ApoE^−/−^/LDLR^−/−^ (*n* = 7) and WT (*n* = 5) mice. Values were presented as mean ± SEM. **P* < 0.05, ***P* < 0.01, ****P* < 0.001 vs WT mice.

**A**


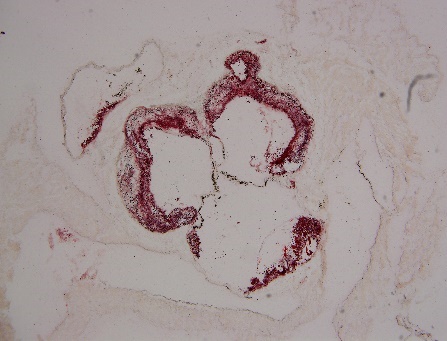

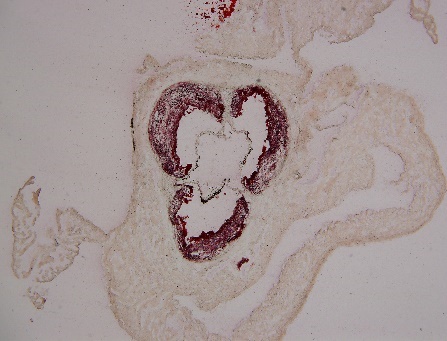

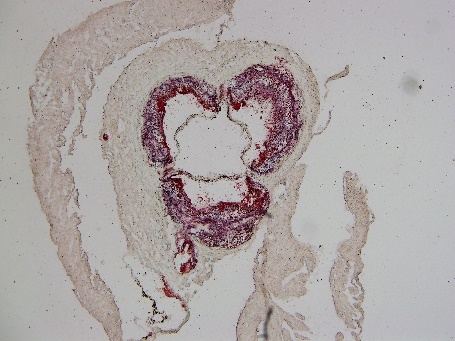


AIN-93G

WD

LCHP

**B C**

**
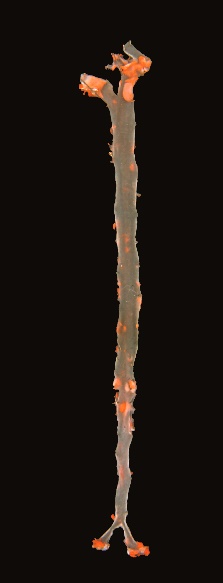
** **
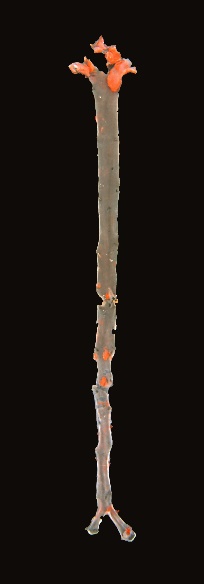
** **
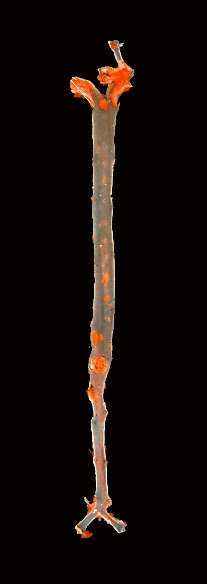
**

**LCHP**

**WD**

**AIN**

**Supplementary Fig. 3.** *Effects of pro-atherogenic (Western (WD) and low-carbohydrate, high-protein (LCHP)) diets on the progression of atherosclerosis and survival of ApoE^−/−^/LDLR^−/−^ mice*. **(A)** Representative images of cross-sections of aortic roots showing aortic plaque stained with ORO or **(B)** en face ORO-stained thoracic aortas in mice fed: Control (AIN-93G), WD or LCHP diet. (**C**) Survival of mice fed: Control (*n* = 42), WD (*n* = 49) and LCHP (*n* = 43) diets. Median survivals: AIN-93G – 10.5 months; WD – 14 months; and LCHP – 6 months. The log-rank *P* values for compared Kaplan-Meier survival curves are as follows: (1) *P* < 0.0001 for the comparisons: AIN-93G vs WD vs LCHP, AIN-93G vs LCHP, and WD vs LCHP, (2) *P* = 0.4687 for the comparison: AIN-93G vs WD.

**Supplementary Fig. 4.** *Significantly changed proteins under familial hypercholesterolemia conditions (validation cohort).* Quantification of selected marker candidates (CRP, PCSK9, ApoC-III, sICAM-1, AGT, PON-1, FETUB, VKDP-S, and BGN) in crude serum samples of FH cases (*n* = 29) and healthy subjects (*n* = 29) by colorimetric ELISA. Protein levels are presented as mean ± SEM. **,* ***,* and *** indicate *P* < 0.05, *P* < 0.01, *P* < 0.001, respectively. *FH+S* – FH patients receiving statin therapy.

**A** **B** **C****D**

**Supplementary Fig. 5.** *Significantly changed serum metabolites under familial hypercholesterolemia conditions (validation cohort)*. The top (**A**) row depicts serum concentration of substrates for NOSs (L-Arg, H-Arg) and Arg precursors (L-Cit, L-Orn), the middle rows -methylated Arg derivatives (NMMA, SDMA, ADMA) and L-Arg/ADMA ratio (**B**), and L-Met, Hcy (**C**), respectively and  **D** refers to other altered metabolites determined in FH cases (*n* = 29) versus healthy subjects (*n* = 29). Data represent mean ± SEM. **P* < 0.05, ***P* < 0.01, ****P* < 0.001. *FH+S* – FH patients on statins.
